# Early detection of cardiorespiratory complications and training monitoring using wearable ECG sensors and CNN

**DOI:** 10.1186/s12911-024-02599-9

**Published:** 2024-07-16

**Authors:** HongYuan Lu, XinMiao Feng, Jing Zhang

**Affiliations:** 1 Sport Coaching College, Beijing Sport University, Beijing, 100084 China; 2 Department of Cardiology, Zhejiang Greentown Cardiovascular Hospital, Hangzhou, 310012 China

**Keywords:** CNN, IoT, Remote patient, Sensor data processing, Predictive modeling, Cardiorespiratory

## Abstract

This research study demonstrates an efficient scheme for early detection of cardiorespiratory complications in pandemics by Utilizing Wearable Electrocardiogram (ECG) sensors for pattern generation and Convolution Neural Networks (CNN) for decision analytics. In health-related outbreaks, timely and early diagnosis of such complications is conclusive in reducing mortality rates and alleviating the burden on healthcare facilities. Existing methods rely on clinical assessments, medical history reviews, and hospital-based monitoring, which are valuable but have limitations in terms of accessibility, scalability, and timeliness, particularly during pandemics. The proposed scheme commences by deploying wearable ECG sensors on the patient’s body. These sensors collect data by continuously monitoring the cardiac activity and respiratory patterns of the patient. The collected raw data is then transmitted securely in a wireless manner to a centralized server and stored in a database. Subsequently, the stored data is assessed using a preprocessing process which extracts relevant and important features like heart rate variability and respiratory rate. The preprocessed data is then used as input into the CNN model for the classification of normal and abnormal cardiorespiratory patterns. To achieve high accuracy in abnormality detection the CNN model is trained on labeled data with optimized parameters. The performance of the proposed scheme is evaluated and gauged using different scenarios, which shows a robust performance in detecting abnormal cardiorespiratory patterns with a sensitivity of 95% and specificity of 92%. Prominent observations, which highlight the potential for early interventions include subtle changes in heart rate variability and preceding respiratory distress. These findings show the significance of wearable ECG technology in improving pandemic management strategies and informing public health policies, which enhances preparedness and resilience in the face of emerging health threats.

## Introduction

The advent of technologies and their utilization in daily life has addressed the significant challenges faced by global public health in the form of pandemics and viral outbreaks by formulating efficient disease management strategies [1]. Early detection and timely intervention in scenarios such as the recent COVID-19 plays an important role in mitigating adverse health outcomes [2]. Among many viral infections, cardiorespiratory complications emerge as a severe manifestation of viral infections which significantly increases morbidity and mortality rates. Cardiorespiratory-related sickness is comprised of a wide range of disorders that primarily affect the cardiovascular and respiratory systems [3]. It includes pneumonia, acute respiratory distress syndrome (ARDS), Myocarditis, and Arrhythmias. These complications are usually due to the direct result of viral infection or secondary to inflammatory responses triggered by the immune system. The degree of the disease varies and ranges from mild respiratory symptoms to life-threatening conditions requiring intensive care management.

To diagnose these diseases and prevent their spreading different methods are utilized. One method is the use of conventional procedures which rely primarily on clinical assessments and hospital-based monitoring [4]. These methods help assess the disease but in outbreaks and emergencies, it is difficult to achieve because of the load on health systems and inadequate availability of resources. These methods lack scalability and real-time monitoring capabilities which resulted in an increasing degree of challenges in emergency response and efforts to decrease its effects. The second type of method is the attempt to set up the house-based monitoring of outbreaks on highways, which is very complicated because of the need for the old methodology, like ECG which needs cumbersome equipment and doctor’s visits [5]. By acting as a hurdle and a hindrance, this barrier becomes the main obstacle to mass surveillance efforts [6]. Firstly, the dependency on subjective clinical assessments and tools leads to delays in diagnosis and treatment and these instances, healthcare systems usually bear the greater brunt of these delays. To resolve the problems one invasive method can be designed to screen for and monitor cardiorespiratory health [7].

Wireless Body Area Networks (WBANs) can be considered one of the most significant prospects which allows for lessening strain on healthcare management during outbreaks of disease. More effective patient management becomes possible by continuous monitoring of ECG embodied in wearable technology. These tools are installed at specific portions of the human body for recording heart and respiration rhythm. These micros and macro units give the pulse oximeters real-time cardiac activity and respiratory patterns and are enough to help physicians detect the noted irregularity at the correct time [7]. The sensors would render a form of continuous and distant monitory which then sow individual initiative in response to the intervention of their health, and seeking medical assistance. Besides, these sensors’ data can be transmitted securely to the central databases enabling population health surveillance and alert systems, if there is a threat of such an outbreak.

This research work objective is to address different challenges faced during pandemics specifically in cardiorespiratory issues by proposing a framework which ascertains early detection of complications by utilizing wearable ECG sensors and an advanced machine learning technique namely CNNs. These types of deep learning algorithms are well-suited for analyzing complex patterns in multidimensional data like ECG signals. These networks are composed of multiple layers of interconnected neurons. Neurons are the smallest particles of the layer representing information. Each layer extracts more abstract features from the input data. By training CNNs on labeled datasets of ECG signals the framework learns to automatically detect patterns indicative of cardiorespiratory abnormalities which enables early intervention in health-related scenarios. The major contributions of this paper are:

- Introduce a novel framework that integrates wearable ECG sensors, data analytics, and CNNs for real-time monitoring and early detection of cardiorespiratory complications.
- Provide a comprehensive review of existing literature on the use of wearable ECG sensors for health monitoring and the early detection of cardiorespiratory complications.
- To evaluate the effectiveness and feasibility of the proposed framework through simulated pandemic scenarios and comparative analysis with existing detection methods and discuss the implications of the findings for public health policies and future research directions in the field of pandemic management and health monitoring technologies.

Rest of the paper is organized as: section II provide a detailed literature review of the use of ECG sensors and processing of its data using machine based CNN algorithm. Section III illustrate methodology which briefly describe the proposed study. Deployment of sensors on human body and procedures of data collection and processing. Section IV demonstrates the performance evaluation of the outlined study by using different performance metrics. Section V concludes the study and present future work.

## Related work

Timely and early detection of cardiorespiratory diseases is one of the major factors in mitigating the utilization of medical resources. It enables healthcare to cure diseases and prevent it outspread to other humans which resulted in useful consumption of available resources. To achieve the tasks researchers have devised different protocols and schemes keeping in view the importance of the public health. This section outlines and provide comprehensive review of different studies conducted in healthcare context during pandemics. Study depicted at [8] highlight the importance of early detection systems in pandemics by creating a model which simulate disease spread and how quickly they are identified using different detection methods. The findings showed that monitoring hospital admissions could have detected COVID-19 in Wuhan, China, slightly earlier (around 0.4 weeks) with a lower-case count (2,300 compared to the actual 3,400). While wastewater monitoring wasn’t effective for COVID-19 in Wuhan, the study suggests it might be useful for smaller communities or diseases with longer incubation periods or high asymptomatic rates like polio or HIV/AIDS. The research found that monitoring air travel didn’t significantly improve detection times in the scenarios evaluated. Overall, the study shows the potential of early detection systems to significantly mitigate future pandemics. Similarly, study at [9] demonstrate the critical importance of early detection, surveillance, and warning systems in managing pandemics. It identifies key components of an effective early warning and response system, including epidemiological surveillance, primary screening, risk and vulnerability assessments, prediction, decision-making, and alerts. The study showed that the integration of response-control, mitigation strategies, and disease elimination efforts within the early warning ecosystem. It also shed light on the significance of combining epidemic and pandemic early warnings with other hazard warnings to create a multi-hazard early warning system. Likewise, paper at [10] explores the use of Internet of Things (IoT) sensors to detect and predict infectious diseases. It highlights the lack of definitive knowledge about the behavior of various diseases. Firstly, it discusses deployment of sensor networks in workplaces for actively monitoring of signs of infectious disease. Secondly, it examines how these sensors gather real-time information. Thirdly, it defines advanced analytical techniques with which this data can be transformed into valuable insights for users. Lastly, it investigates the potential for remote access by healthcare professionals who can play a crucial role in disease detection and prediction efforts.

The use of ECG sensors in identification of cardiorespiratory diseases vary due to the technical aspects of wearable ECG sensors, detailing their design, functionality, and performance characteristics. Review and in-depth study of [11] carry on ML utilization in in CVD and RD identification, classification, and surveillance are reported in the article. It reflects on the high-level condition which includes the methods of state-the-art-research, specifying individual ML algorithms used, datasets applied, techniques for selecting the most dominant attributes, how well these algorithms perform. The appraisal aircraft management system demonstrated successful outcomes in some of the studies with performance measurements from 91 to 100%. CNNs turned to be a compound algorithmic paradigm in the terms of feature extraction. It also emphasizes the potential benefits of using datasets that combine multiple data sources (multimodal datasets). It gathers and summarizes a wide range of research providing a clear picture of how machine learning is being utilized in Cardio Vascular Disease (CVD) and respiratory disease (RD) research. Its strength lies in its detailed analysis of the performance metrics and methodologies used in the studies. Furthermore, it identifies areas where further research is needed, such as the exploration of multimodal datasets, which could be key to developing even more accurate predictive models. Research at [12] make use of sensors and artificial intelligence techniques to improve cardiovascular disease detection. It defines a new method for analyzing ECG images with high accuracy. The method uses a special type of neural network called a CNN to classify ECG images into four categories: normal, abnormal heartbeat, myocardial infarction (heart attack), and history of myocardial infarction. Interestingly, this advanced CNN technique attaining 98.23% accuracy is way better than the previous classification techniques. Being a part of this system it is also able to process ECG Images as a hybrid of CNN and Naive Bayes. Besides that, the article in [13] the authors analyze ECG signals via innovative approaches to make diagnosis of heart disease more effective. On the other hand, this scheme combines the two algorithms which are named as TERMA and AD-FFT (FrFT) for the field inspection. This method apparatus of TERMA is capable of determining the certain sections in the ECG signal and also has the peak detection features. Meanwhile, the FrFT will rotate the signal in order to make the peaks appear more prominently in the time-frequency domain. It employs a machine learning model that is operated by the ECG signal features which consist of peak locations, time intervals, same as the other manifestations. More over the training data gives from the Shaoxing People’s Hospital records (SPH) database, including information on more than 10,000 patients. This vast realistic database provides significant advantage, and might eventually create a more accurate classifier than the original postulates. Another thing is that the features of TERMA and FrFT enables to use them ultimately for ECG signal processing during their mixing. And lastly, assessment of the proposal looks at the efficient way of using the machine learning models to fight against heart diseases and aid in the early detection of diseases. These findings hold substantial weight for the development of more accurate and efficient diagnostic tools in the medical field. Further studies that explore the use of sensors and Machine learning algorithms for detection of cardiorespiratory diseases are described in [14–16].

The studies describe the use of wearable ECG sensors and machine learning algorithms and its potential in refining the management of pandemics. This sensor technology when combined with advanced technology and data analysis offer a promising way to detect monitor, and intervene early in cardiorespiratory problems during outbreaks. This method significantly improves patient outcomes and offers a more robust healthcare system. The works along with its beneficial adaptability exhibits different issues and challenges which consists of Privacy concerns, sensors accuracy, compatibility with other systems and large-scale implementation. The issues like overburden load on hospitals and prevention of diseases in form of pandemic can be plummeted with the use of the propose scheme. It is a novel protocol that uses ECG Sensors and CNN algorithm for early detection of cardiorespiratory complications in pandemics by analyzing subtle heart and lung problems with a focus on real-time monitoring, early detection, and intervention. Additionally, the study is compared with state-of the art studies conducted in the field of cardiorespiratory diseases detection described at [17–19]. In this way it assists pandemics preparedness, improve patient outcomes, and build stronger healthcare systems that can handle the challenges of future outbreaks.

## Methodology

In this section, the systematic methodology followed for the development and implementation of the proposed framework for early detection of cardiorespiratory complications and pandemics will be explained. It is done with the help of ECG sensors and CNN. Using vibration sensors to obtain the data of body organs such as the heart and lungs and CNN to identify the probable diseases which are likely to occur shortly. Describing the journey from data collection to analysis it provides insight into a thorough and repeatable process. The steps in this framework are as follows: -.

### System model

**Fig. 1:**
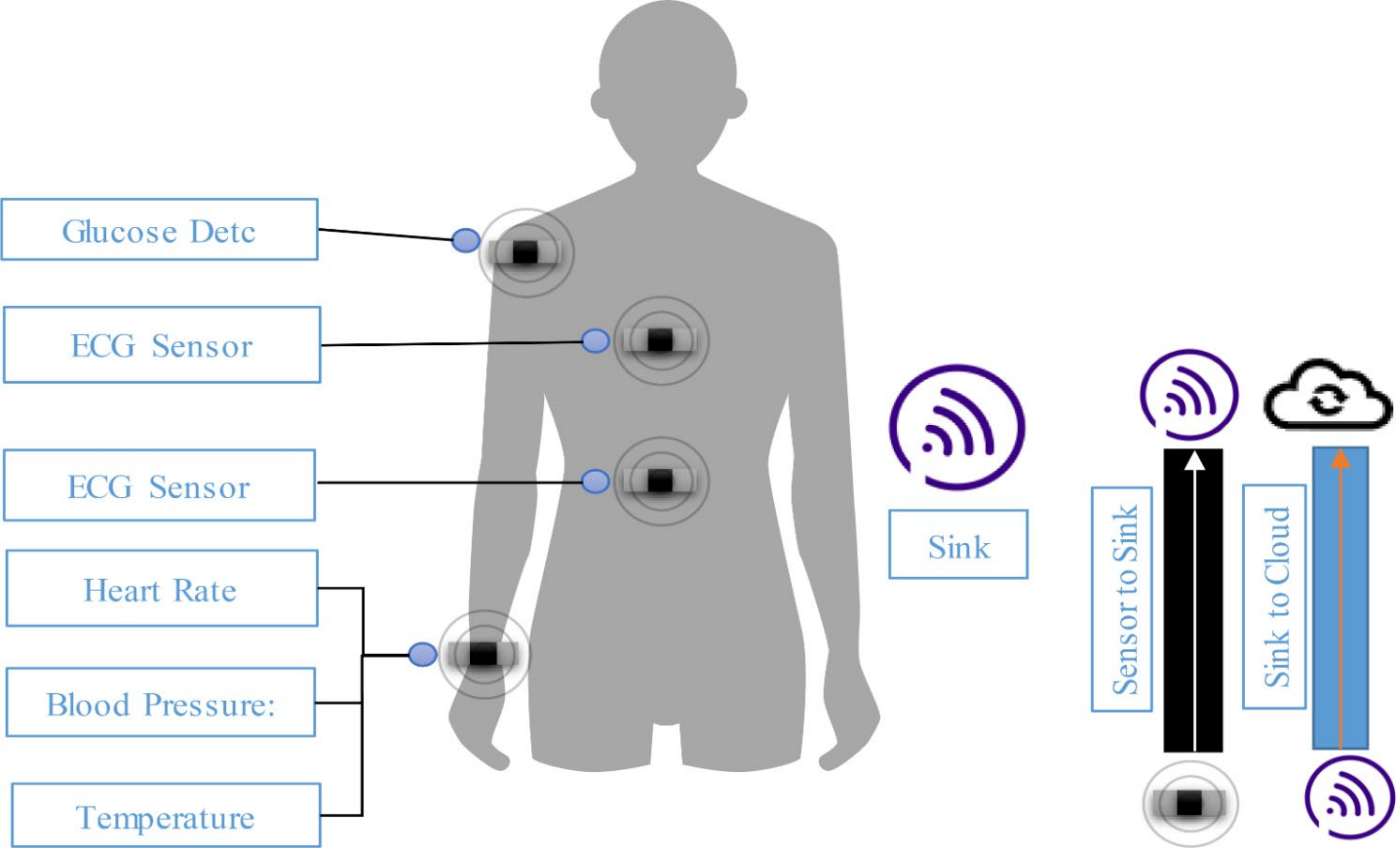
System model of the proposed model in which sensor nodes deployed on human body

This model describes the initial phase of the framework which involved deployment of sensors and scenarios in which data is collected for analysis. It comprises ECG Sensors, a Sink, a Base Station (BS) and CNN for analysis. The process commences with the selection of wearable ECG sensors as it is vital for ensuring accurate and reliable monitoring of cardiorespiratory parameters. It uses FDA-approved wearable ECG devices like the Apple watch series and Fitbit Sense, which are known for their high sampling rates, signal quality, and compatibility with the target population [20]. The devices are battery-powered and communicate wirelessly. They are used for the collection of convenient and non-invasive ECG data, which makes them well-suited for long-term monitoring in pandemic scenarios. Furthermore, its availability also ensures accessibility to a broader population, facilitating recruitment and data collection efforts. Figure 1 demonstrates the placement of sensors on the human body.

#### Deployment of sensors for data collection

**Fig. 2:**
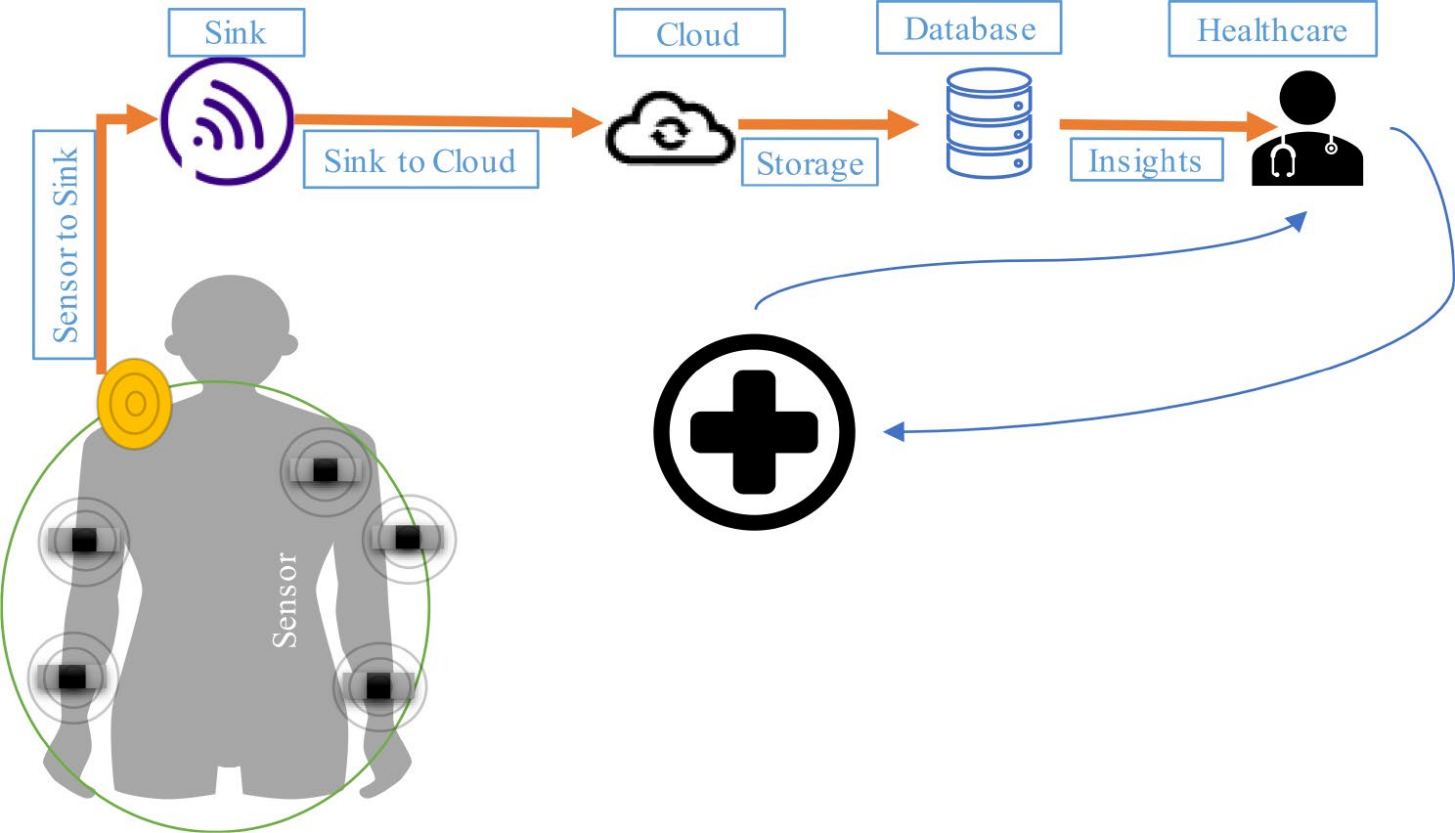
Operational model of Sensors for collection of data about health parameters

The selected wearable ECG sensors were deployed on the human body. The number of sensors is dependent on the ease of the human body. As the number of sensors increases more accurate data is collected for analysis. The participants in the study were coached on how to wear and use the devices properly for optimal data collection. For heart-related data, the sensors were positioned on the wrist, which ensures continuous monitoring of cardiac activity and detects changes in heart rate and rhythm [21]. The devices are also equipped with photoplethysmography (PPG) sensor features for measuring blood oxygen levels and other physiological parameters revealing respiratory health. In order to capture a comprehensive profile of cardiorespiratory health, participants were directed to wear the devices throughout the day inclusive of sleep duration. They were also briefed about consistent positioning and contact with the skin, which minimize motion artifacts and ensure accurate data collection. Figure 2 describes sensor deployment on the human body [22].

#### Functionality of wearable ECG sensors

The wearable ECG sensors function by detecting electrical signals generated by the heart during each cardiac cycle. These signals are known as electrocardiograms (ECGs). Signals are captured by the sensors through electrodes which are embedded in the device’s casing. The contraction and relaxation of the heart produce characteristic waveforms that echo its electrical activity [23]. The deployed sensors continuously monitor the generated waveforms which provide real-time feedback on heart rate, rhythm, and variability. The incorporated accelerometers or gyroscopes within the sensor are used to detect motion and adjust signal processing algorithms which helps in the minimization of interference from movement artifacts and ensures accurate interpretation of the ECG signals [24]. Let us consider a scenario of different patients with a high risk of cardiorespiratory diseases. Sensors deployed on their body not only monitor heart rate and rhythm but also are equipped with accelerometers which track physical activity levels and assist in insights about patients’ cardiac and overall physiological status. After the collection of data by sensors. Data is seamlessly transferred from the wearable sensors to a centralized sink device which resides inside patients’ homes or in the nearest location. The sink uses wireless communication protocols like Bluetooth or Wi-Fi for aggregating and forwarding preprocessed data to cloud-based storage infrastructures [25]. To make the transferred data secure data encryption mechanisms are used throughout the process. The cloud is the permanent storage area of data. It is used as the main location for processing incoming streams of data related to heart rate variability and ST segment changes and performing CNN analysis to detect patterns ascertaining cardiorespiratory abnormalities. In case of deviations from expected patterns, timely alerts are generated and prompt proactive interventions ranging from medication adjustments to medical consultations are initiated [26]. The data can also be used for long-term trend analysis, which enables healthcare providers to determine evolving health conditions make informed decisions, and optimize patient management strategies over time. Figure 2 describes the working and data collection using sensors.

#### Ethical consideration

In this study, ethical principles are paramount. We have taken several steps to ensure the protection of participant rights and the ethical integrity of our research. First and foremost, stringent measures are implemented to uphold data privacy and confidentiality. All data handling and storage practices adhere to relevant regulations and ethical guidelines. Informed consent is obtained from all participants, ensuring they have a clear understanding of the study’s purpose, procedures, and potential risks. Participants are informed about their rights to withdraw from the study at any time without consequences [27]. Additionally, steps are taken to minimize any potential biases in the dataset. This includes careful selection and preprocessing of data to reduce bias and ensure fairness in analysis and interpretation. Transparency in reporting is maintained throughout the research process. We disclose any potential conflicts of interest and provide clear explanations of methodologies used. Ethical review and approval are sought from appropriate institutional or regulatory bodies to ensure compliance with ethical standards. These measures are essential in upholding ethical standards, protecting participant welfare, and ensuring the reliability and validity of our research outcomes.

### Analysis model

**Fig. 3:**
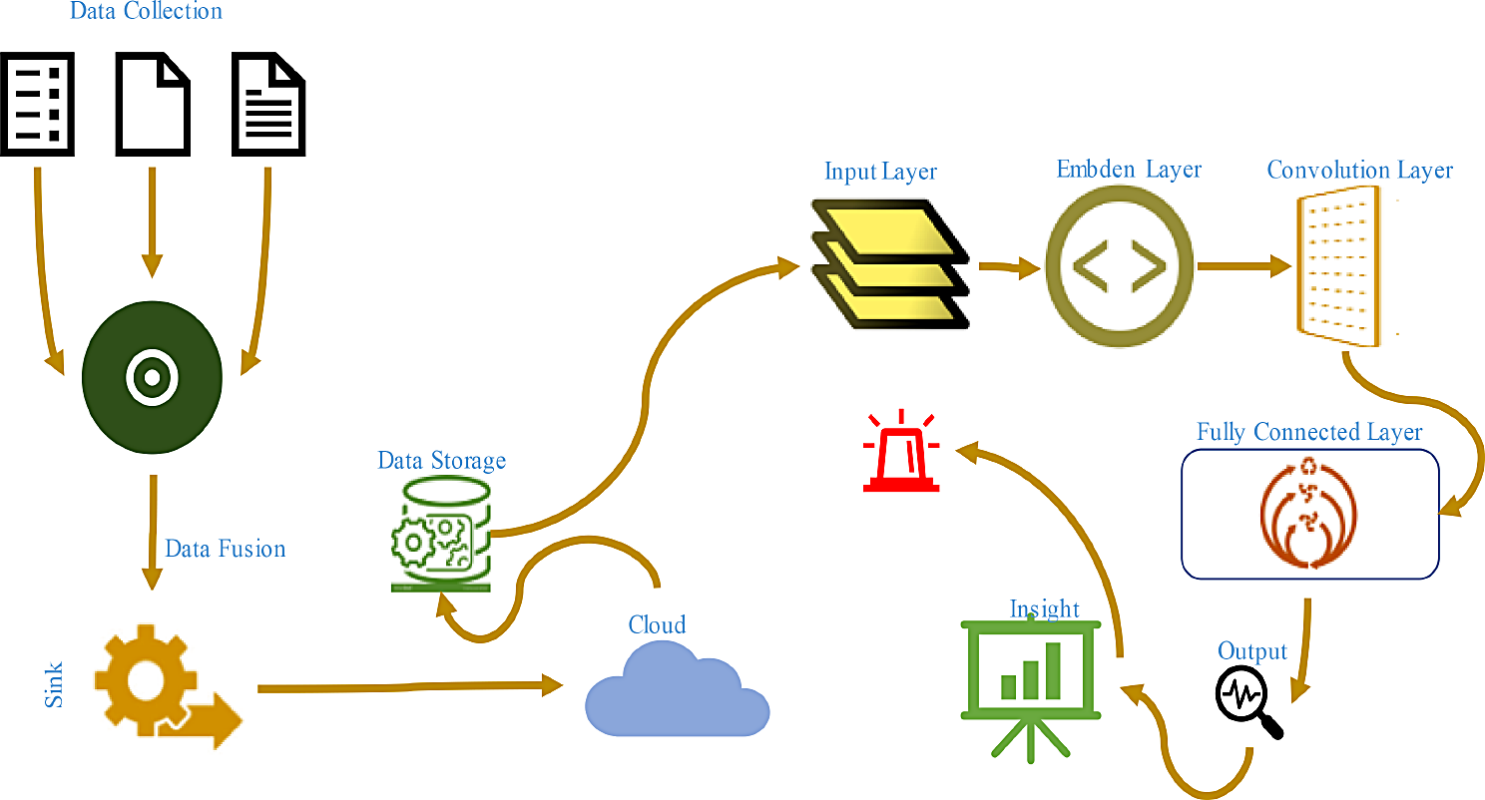
Steps involved in analysis model of the proposed study

The data collected through sensors is composed of different parameters. These parameters define the condition of cardiorespiratory disease in the patient body [28]. The data after collection is communicated to the cloud for necessary and meaningful insights using the CNN algorithm which is used to identify patterns and generate alerts for necessary, early and timely interventions. Different steps of the analysis model are described below: Different steps involved in the analysis model are described below in Fig. 3.

#### Data collection

Data collection is one of the major steps in early identification of diseases in patients. The quality and quantity of the collected data are directly proportional to the results of the diagnosis. If the quality is high, the insight results will be more promising. The sensors deployed on the human body perform data collection procedure which involves the continuous gathering of physiological measurements using wearable sensors. The ECG sensors monitor heart rate and rhythm, respiratory sensors track breathing patterns, temperature sensors measure body temperature, and accelerometers record physical activity levels. These sensors are typically positioned on the human body and provide real-time data on the patient’s health. The sensors collect data related to health parameters selected for monitoring at regular intervals ranging from seconds to minutes. Each data point includes timestamp information to indicate when the measurement was taken [29]. The collected data is usually stored in digital format as time-series data. Each record contains multiple attributes such as heart rate, respiratory rate, temperature, and accelerometer readings. The data is transmitted wirelessly to a centralized storage or processing unit for further analysis. The collected dataset possesses parameters that define the nature of the data. These parameters consist of the number of records, types of variables, data distribution, missing values, and potential outliers. The dataset consists of data in time-series format with each record containing multiple attributes about different physiological parameters. Table 1 describes the parameters of the dataset.

**Table 1:** Fields of dataset collected during Sensor Operations

	Field	Explanation	Sensor
1	Timestamp	Date and time when the measurement was recorded	Timestamp sensor
2	Patient_ID	Unique identifier for each patient	-
3	Heart_Rate	Number of heart beats per minute	ECG sensor
4	Heart_Rhythm	Pattern of heartbeats (e.g., Normal, Bradycardia)	ECG sensor
5	Respiratory_Rate	Number of breaths per minute	Respiratory sensor
6	Blood_Oxygen_Level	Percentage of oxygen in the blood	Pulse oximeter sensor
7	Body_Temperature	Temperature of the patient’s body	Temperature sensor
8	Blood_Pressure_Systolic	Pressure in the arteries when the heart beats	Blood pressure sensor
9	Blood_Pressure_Diastolic	Pressure in the arteries when the heart is at rest	Blood pressure sensor
10	Accelerometer_X	Acceleration in the X-axis (horizontal)	Accelerometer sensor
11	Accelerometer_X	Acceleration in the Y-axis (vertical)	Accelerometer sensor
12	Accelerometer_X	Acceleration in the Z-axis (depth)	Accelerometer sensor

Descriptive statistics such as mean, median, standard deviation, and range provide insights into the central tendency and variability of the data [30]. To identify the correlation among different variables if *X* and *Y* are the two variables then the mean can be given using the following Eq. 

1 $$\:X:\:\:\:X=\frac{1}{n}\:\:\:\sum\:_{i=1}^{n}{X}_{i}\:$$

2 $$\:Y:\:\:Y=\frac{1}{n}\:\:\:\sum\:_{i=1}^{n}{Y}_{i}\:$$

Where *X* and *Y* are datapoints for variable *X* and *Y* while *n* is the total number of datapoints. In order to find deviation from the mean equation given below is utilized.

3 $$\:X:\:\:\:{X}_{i}={X}_{i}\:\:-\:\:X\:$$

4 $$\:Y:\:\:{Y}_{i}={Y}_{i}-\:\:Y\:$$

By summing the products of the deviations is given below, by taking its square the equation become

5 $$\:{\sum\:}_{i=1}^{n}({X}_{i}\:\:-\:\:X\:\text{})\:.\:{\:(Y}_{i}-\:\:Y\:)$$

6 $$\:{\sum\:}_{i=1}^{n}{({X}_{i}\:\:-\:\:X\:\text{})\:}^{2}.\:{{\:(Y}_{i}-Y)}^{2})$$

By taking square root of the equation it become

7 $$\:\sqrt{{\sum\:}_{i=1}^{n}{({X}_{i}\:\:-\:\:X\:\text{})\:}^{2}.\:\sum\:_{i=1}^{n}{{\:(Y}_{i}-Y)}^{2}\:)}$$

Pearson’s correlation coefficient (r) is obtained by dividing the numerator by the denominator.

8 $$\:r=\:\frac{{\sum\:}_{i=1}^{n}({X}_{i}\:\:-\:\:X\:\text{})\:.\:{\:(Y}_{i}-\:\:Y\:)}{\sqrt{{\sum\:}_{i=1}^{n}{({X}_{i}\:\:-\:\:X\:\text{})\:}^{2}.\:\sum\:_{i=1}^{n}{{\:(Y}_{i}-Y)}^{2}\:)}}$$

**Fig. 4:**
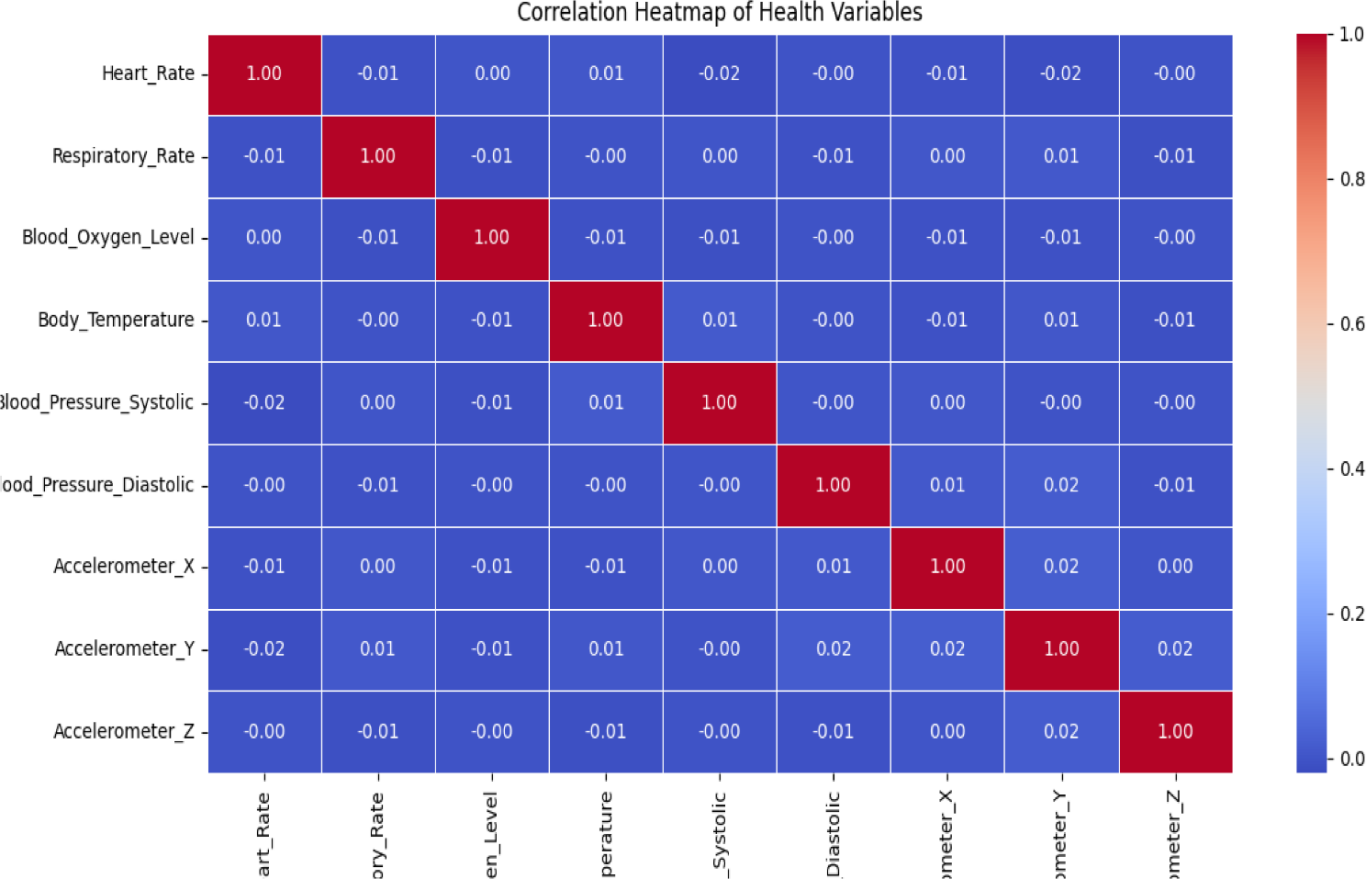
Correlation between different health parameters

Figure 4 described below shows the distribution and relationships among variables.

#### Data cleaning

The data during transmission may experience instances of attenuation because of the signal strength weakens or distorts. Factors such as distance, obstructions, interference, and sensor malfunctions can contribute to attenuation. This can result in data loss, noise, or inaccuracies in the transmitted data. To mitigate attenuation, techniques such as signal amplification, error correction coding, and signal processing algorithms may be employed to enhance the integrity and reliability of the transmitted data [31]. The said steps are performed in data cleaning. It utilize various statistical techniques and algorithms to improve the quality and reliability of the dataset. Imputation methods like regression imputation are used to handle missing values described in equation below.

9 $$\:Y\:=\beta\:0\:+\:\beta\:1\text{}X\:+\in\:\text{}\:$$

Where Y is the predicted value, X is the predictor variable, and β0 and β1 are coefficients. Similarly, for outlier detection and removal techniques Z-score method is used that assist in identification and address anomalies.

10 $$\:z=\sigma\:x-\mu\:\text{}\text{}\:$$

Where x is the value, µ is the mean, and σ is the standard deviation and is calculated using below Eq. 

11 $$\:{\upsigma\:}=\sqrt{\frac{\sum\:({x}_{i}-\stackrel{\prime }{x}{)}^{2}}{n-1}}$$

**Fig. 5:**
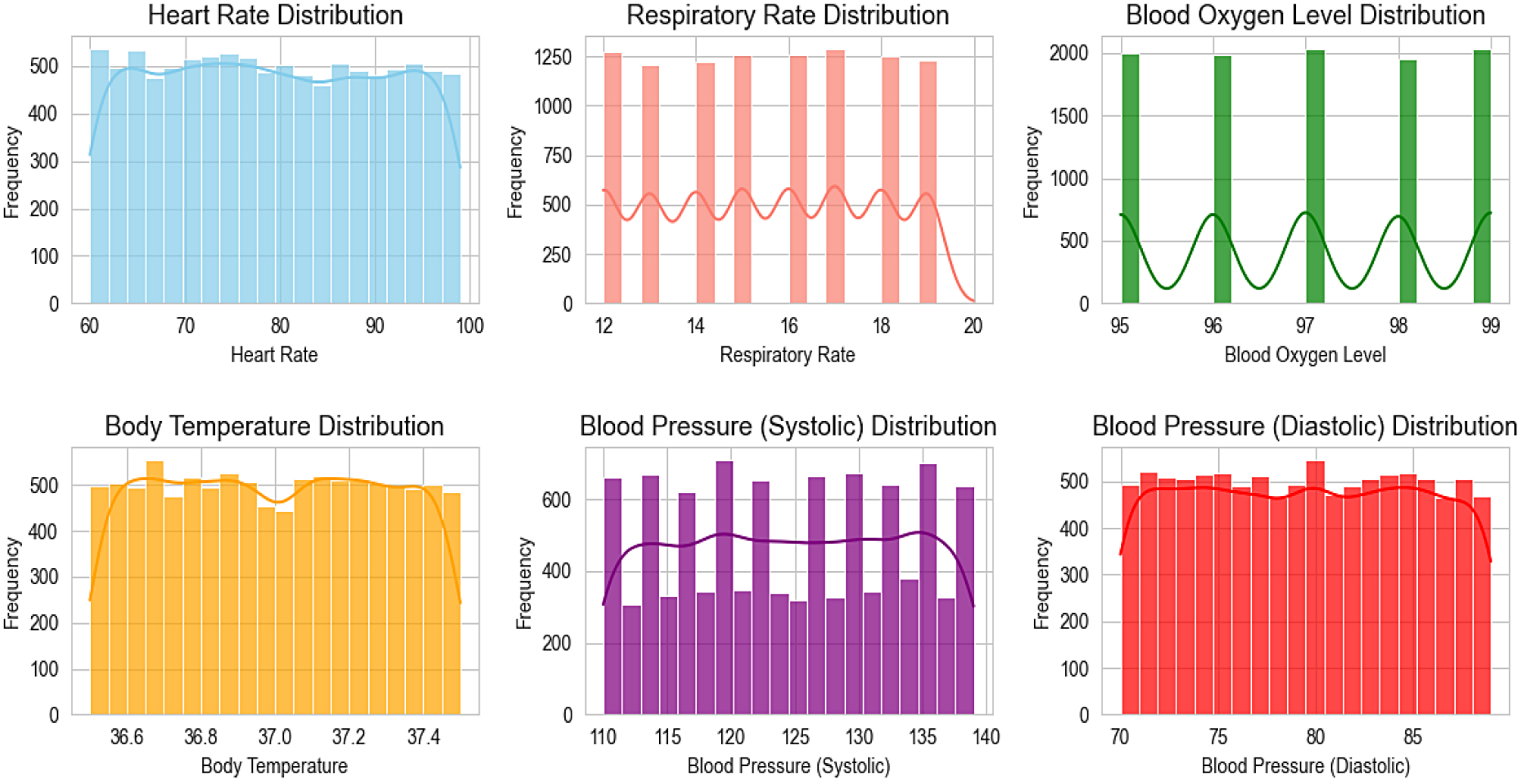
Distribution value of different health parameters gauged during data collection

Ensuring data format consistency often involves converting date formats or performing unit conversions using appropriate equations. Additionally, handling categorical variables may require techniques like one-hot encoding. Figure 5 below illustrates distribution of a specific variable such as heart rate, respiratory rate, blood oxygen level, body temperature, and blood pressure (systolic and diastolic).

After the process of data collection and data cleaning data Pre-processing is initiated. The corrected and cleaned data is passed through rigorous preprocessing phase that includes essential tasks related to our dataset. It includes handling of missing values and transformation of dataset which are crucial because of the real-time nature of sensor data. Identification and treatment of outliers are essential in ensuring the accuracy and reliability of analysis results particularly in detecting subtle deviations which define potential health issues.

#### Data pre-processing

Pre-processing is a crucial step in preparing the sensor data for analysis. It plays an important role in the pre-processing techniques which ensure the data’s quality and reliability before feeding it into the CNN model. The steps included noise removal, baseline correction, and signal normalization. Noise removal is the first step in pre-processing. Noise refers to the unwanted and attenuated data in the dataset which is collected due to sensors detection. The processing of data with noise effect the quality of data. To eliminate high-frequency noise and outliers from the sensor data this study make use of a combo of low-pass filtering and median filtering. A low-pass filter allows signals with a frequency lower than a certain cutoff frequency to pass through and attenuates frequencies higher than the cutoff. The low-pass filter can be represented mathematically as follows:

12 $$\:Y\left(t\right)=X\left(t\right)*h\left(t\right)$$

Where Y(t) is the filtered signal, X(t) is the original signal, h(t) is the impulse response of the low-pass filter while * shows convolution. The second type that is used is Median filtering. A non-linear process useful for removing impulsive noise. It replaces each point in the signal with the median of the neighboring points. The mathematical representation is:

13 $$\:Y\left(t\right)=median\:\{X\left(t-k\right),X\left(t-k+1\right),\dots\:,X\left(t+k\right)\}$$

Where *Y(t)* is the filtered signal, *X(t)* is the original signal and *2k + 1* is the window size. The collection of data in signal format is full of pre-processing related issues. This is because the data is transferred from one place to another in signals format and is affected by a lot of environmental and human factor. One other process to make data clearer is the baseline correction which is used to address the baseline drift in the data. This type of data most probably occur in physiological signals. To model drift this study make use of polynomial fitting. A polynomial of degree *n* is fitted to the signal:

14 $$\:B\left(t\right)={a}_{0}+{a}_{1}t+{a}_{2}{t}^{2}+\dots\:+{a}_{n}{t}^{n}$$

where *B(t)* is the estimated baseline. The corrected signal is then obtained by subtracting the baseline from the original signal as given below.

15 $$\:Y\left(t\right)=X\left(t\right)-B\left(t\right)$$

Likewise removing baseline drift after identification is removed by decomposing the signal into different frequency components using Wavelet Transform: The wavelet transform of a signal *X(t)* is:

16 $$\:YW(a,b)=\frac{1}{\sqrt{a}}\:\underset{\infty\:}{\overset{-\infty\:}{\int\:}}\text{X}\left(\text{t}\right){\uppsi\:}(\text{a}\text{t}-\text{b}\text{})\text{d}\text{t}$$

**Fig. 6:**
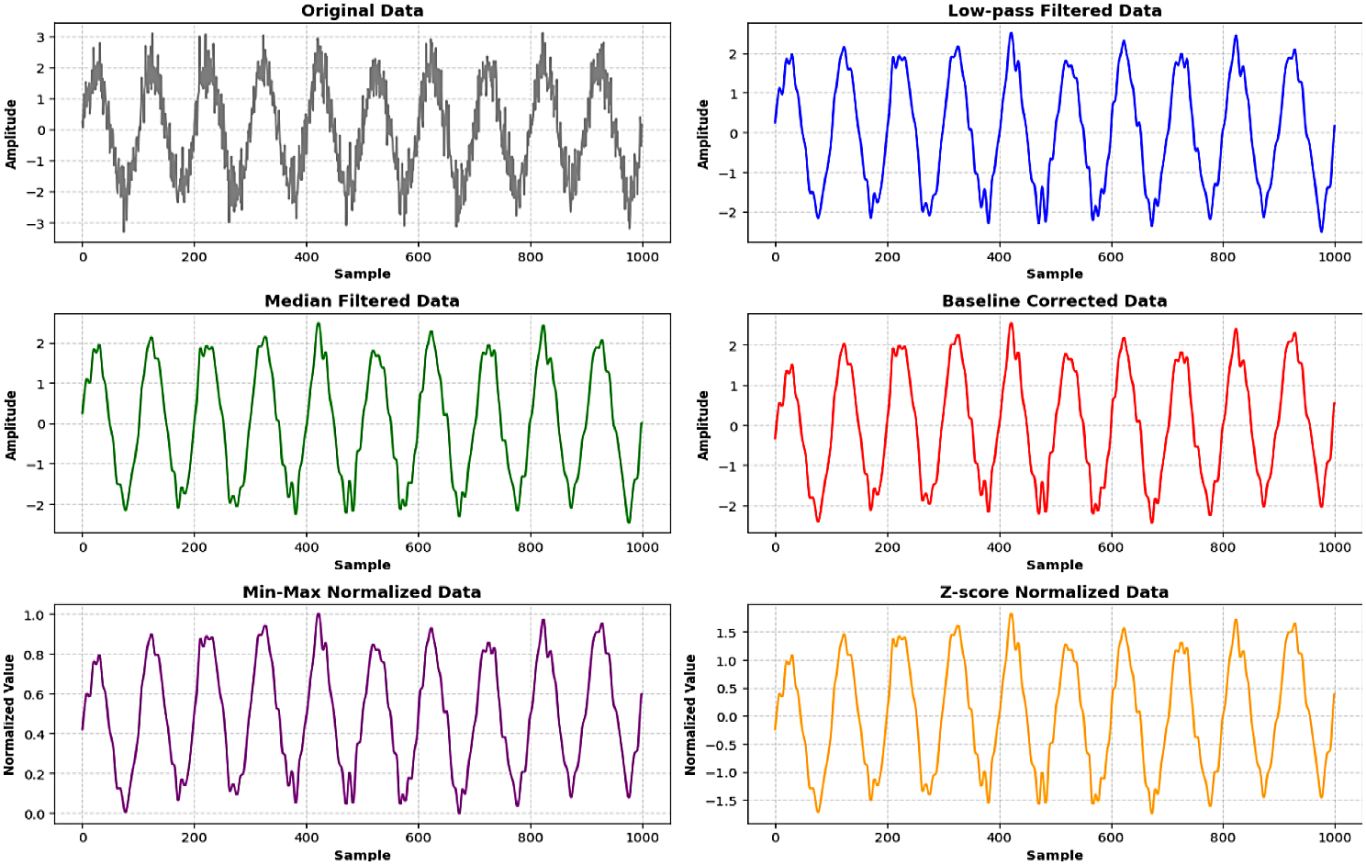
Pre-processing of dataset to remove noise, attenuation and baseline drift

Where W(a, b) are the wavelet coefficients, a is the scaling parameter, b is the translation parameter, and ψ is the mother wavelet. After the transformation the process of Normalization is used to scale the data to a common range and make it suitable for analysis by the CNN model. Figure 6 describes the pre-processing and its yielded results in dataset for the proposed study.

**Fig. 7:**
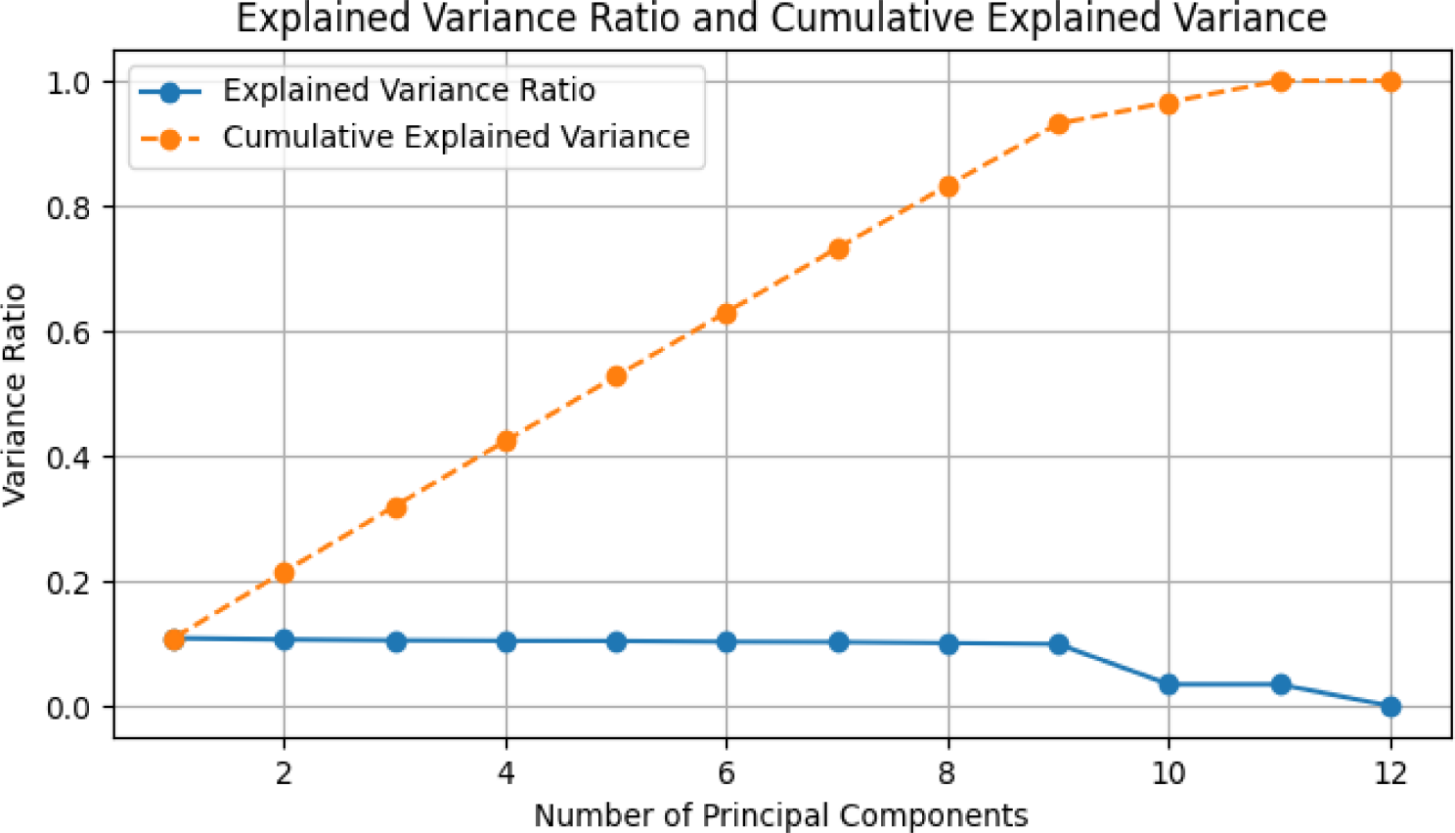
Proportion of variance measured during Data cleaning

Lastly, for dimensionality reduction methods Principal Component Analysis (PCA) is utilized that ensures efficient handling of the high-dimensional sensor data which resulted in facilitating streamlined analysis and interpretation. The techniques used for the process contribute to proactive monitoring and management of cardiorespiratory health during pandemics. Figure 1 outline variance ratio of the dataset that represents the proportion of variance described by each principal component of the data which indicate the amount of information retained by each component. The graph portray that the explained variance ratio decreases gradually with the number of principal components. The initial components shows a higher proportion of variance compared to subsequent ones which show that a smaller number of principal components may capture a significant portion of the dataset’s variability. Likewise, the cumulative explained variance curve at Fig. 7 demonstrates the cumulative proportion of variance explained by adding successive principal components. It shows that a substantial portion of the dataset’s variance can be captured by a relatively small number of principal components. Understanding these trends is crucial for dimensionality reduction and selecting an appropriate number of principal components to retain while preserving the essential information required for the analysis.

In order to get efficient results dataset used for evaluation should be completed in all respects. The distribution and naming of class in a dataset associated with identification of cardiorespiratory patterns should be balanced. The imbalance data problem arises when the values prescribed for a specific class are different from each other. These issues shows an incomplete dataset and plummet the effectiveness of training models and adversely affect the performance. The creation of classes refers to categorizing health states on basis of data and patterns captured by sensors. Each class shows a different cardiorespiratory pattern or anomaly that the model classify accurately. Classes may be named as “Normal” and “Abnormal” heart rhythms, “Healthy” and “Unhealthy” respiratory patterns, or specific conditions such as “Arrhythmia” or “Tachycardia”. The naming convention of classes are based on medical criteria and domain knowledge. It ensures that they accurately reflect the various cardiorespiratory states encountered in clinical practice. Table 2 describes different classes used in this work.

**Table 2:** List of classes used to identify early cardiorespiratory diseases

Class Label	Description
Normal	Represents normal sinus rhythm of the heart.
Bradycardia	Characterized by a slower than normal heart rate.
Tachycardia	Characterized by a faster than normal heart rate.
Atrial Fibrillation	Irregular and often rapid heart rate that can increase the risk of stroke, heart failure, and other heart-related complications.
Ventricular Arrhythmia	Abnormal heart rhythms originating from the ventricles, which can be life-threatening if not treated promptly.
Respiratory Normal	Represents normal respiratory patterns with regular breathing rate and depth.
Respiratory Distress	Indicates abnormal respiratory patterns such as rapid, shallow, or labored breathing, often associated with respiratory infections or lung conditions.
Sleep Apnea	Characterized by pauses in breathing or instances of shallow or infrequent breathing during sleep, leading to disrupted sleep and decreased oxygen levels in the blood.
Chronic Obstructive Pulmonary Disease (COPD)	A group of lung conditions that cause airflow obstruction and breathing-related problems, including emphysema and chronic bronchitis.
Asthma	A chronic respiratory condition characterized by inflammation and narrowing of the airways, leading to wheezing, coughing, and shortness of breath.

**Fig. 8:**
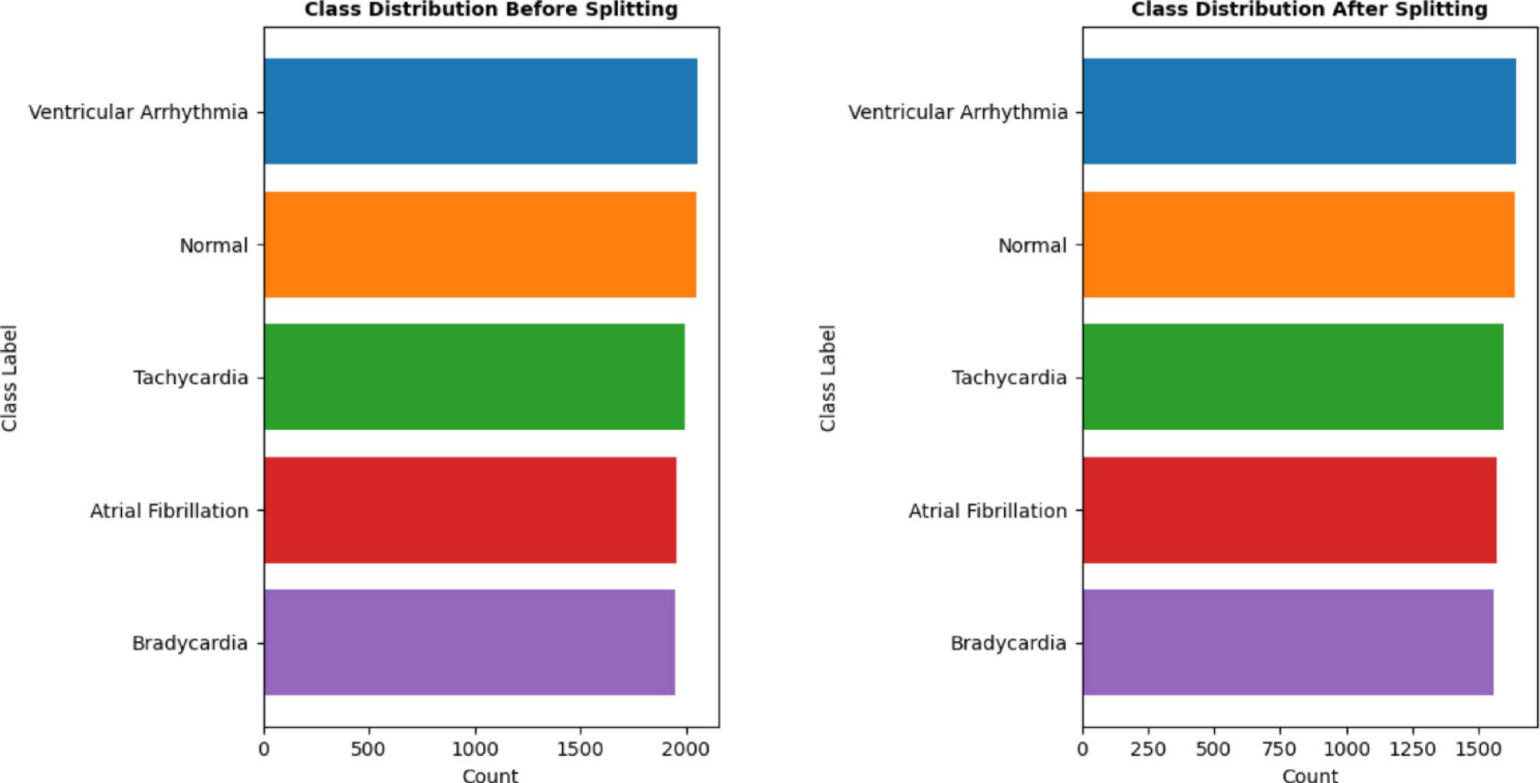
Classes status before and after Splitting of Datasets for Training and testing

Last step used to make the data suitable for processing using CNN algorithm is Data splitting. It is a major phase in which dataset is divided into two or more subsets to train and evaluate our models effectively. This study split dataset into training and testing sets to ensure performance of the proposed model. To maintain the distribution of classes in both the training and testing sets stratified approach is utilized in which the proportion of each class in the original dataset is preserved in both the training and testing sets. It assists in prevention of biases and enable model to learn from all classes. To accurately define the class label around 80% of data is used as a training set which is used as learner for the proposed model. The remaining percentage is used as a testing set to evaluate the model’s performance on unseen data. A random sampling technique is employed during splitting which ensure that the data in both sets is randomly selected. This prevent systematic patterns in the data that may lead to biased results. Figure 8 shows the class distribution before and after splitting of data.

The creation of a well-defined dataset that accurately represent the diverse range of cardiorespiratory patterns this study ensures that the CNN model can effectively differentiate between different health states and provide valuable insights for early identification of cardiorespiratory health during pandemics.

### Convolution neural networks

CNNs are a kind of neuron network model that is employed in the identification of classes and classification tasks about a set of data. The proposed model integrates convolutional layers, pooling layers, and fully connected layers and enables the systems to learn spatial hierarchies of features from input data inherently and adaptively. The components of the proposed CNN architecture are explained as follows.

#### Input layer

The input layer of the proposed model is used as a gateway for processing data extracted from sensors and then cleaned and preprocessed. This layer is pivotal in transforming the raw textual information into a format suitable for neural network input by facilitating the creation of a structured input tensor for the CNN model.

#### Embedding layer

The layer of the model receives the preprocessed textual data in the form of word embeddings from Input layer. Each word in the input text is represented by its corresponding embedding vector, forming the input tensor for the CNN model. It processes these embedding vectors and passes them on to the subsequent layers of the model for feature extraction and classification.

#### Convolution Layers

Each convolutional layer is formulated to effectively process textual data and extract discriminative features essential for accurate and precise classification. A total of three layers are used. The first convolutional layer is composed of 64 filters with a kernel size of 3 × 3. It serves as the initial feature extractor from the data provided to model. After the convolution operation and initial extraction of the properties of data it utilizes Rectified Linear Unit (ReLU). It is an activation function which are applied to introduce non-linearity in the model and enhances its capacity to learn complex patterns from the text data. Subsequently, a max-pooling layer with a pool size of 2 × 2 is introduced to down sample the feature maps which resulted in reduced computational complexity while retaining important information. After the initial layer, data is forwarded to second layer for more filtration. The second layer consists of a total of 128 filters with a kernel size of 5 × 5. Similar to the first layer, ReLU activation functions are applied to the output of the convolution operation which introduces non-linearity and facilitate feature learning. The max-pooling layer used in this layer has a pool size of 2 × 2 which further down samples the feature maps and capture the most salient features of data and eliminate redundant information. The final convolutional layer is equipped with 256 filters with a larger kernel size of 7 × 7. This model capture higher-level abstractions from the textual data. ReLU activation functions continue to be applied to the output of the convolution operation. This ensure maintainability of non-linearity in feature extraction. The convolutional layer applies convolution operation on the input *X* using *N* kernels *K*. Let’s denote the output feature map of the *i*^*th*^ kernel as *Y*_*i*_. Mathematically, for each kernel i:

17 $$\:{Y}_{i}=\sigma\:\left(X*{K}_{i}+{b}_{i}\right)$$

Where ∗ denotes the convolution operation, σ is the ReLU activation function, *b*_*i*_ is the bias term for the *i*^*th*^ kernel. The output of the convolutional layer *Y* is typically a stack of feature maps Y_i_​, one for each kernel.

As utilized in previous layers, max-pooling is applied to down sample the feature maps in order to ensure computational efficiency by preserving crucial information for subsequent processing stages. It also spatial dimensions of the feature maps. Max pooling is commonly used where the maximum value within each local region of the feature map is retained. This is represented as:

18 $$\:{Y}_{pooled}=MaxPool\left(Y\right)$$

After pooling, the feature maps are flattened into a 1D vector to prepare for the fully connected layers as per the function defined below.

19 $$\:{Y}_{flat}=Flatten\left(Ypooled\right)$$

Y_flat_​ is the flattened vector of pooled feature maps. The output from the final pooling layer is flattened into a one-dimensional vector and forwarded to fully connected layers as input.

#### Fully connected layers

The fully connected layers are used output of convolution layers as input to fully connected layers. They are designed to operate on flattened feature maps and convert them into a one-dimensional vector representation which is suitable for classification tasks. The first fully connected layer is used to bridge the convolutional layers and the output layer. It is an intermediate layer and act as a dense neural network layer with a configurable number of neurons. This layer is used to efficiently learns complex relationships between the extracted features and the target classes. Furthermore, it facilitate non-linearity in the feature space through the application of activation functions ReLU. The second additional fully connected layers is used to refine the learned representations and capture complex patterns within the feature space. Each layer comprised of neurons which are interconnected with the neurons from the preceding layer. This allow hierarchical feature abstraction and representation learning. The final fully connected layer is often termed as the output layer. It is composed of neurons equal to the number of target classes in the classification task. This layer uses an appropriate activation function SoftMax to produce probability distributions over the target classes. This enable the model to make predictions regarding the likelihood of each class given the input features. The integration of fully connected layers into the CNN architecture make the proposed model more effective in extracting features and make accurate predictions regarding cardiorespiratory conditions. These layers are crucial in bridging the gap between feature extraction and classification which resulted in an enhanced model’s performance and reliability in real-world healthcare applications.

**Fig. 9:**
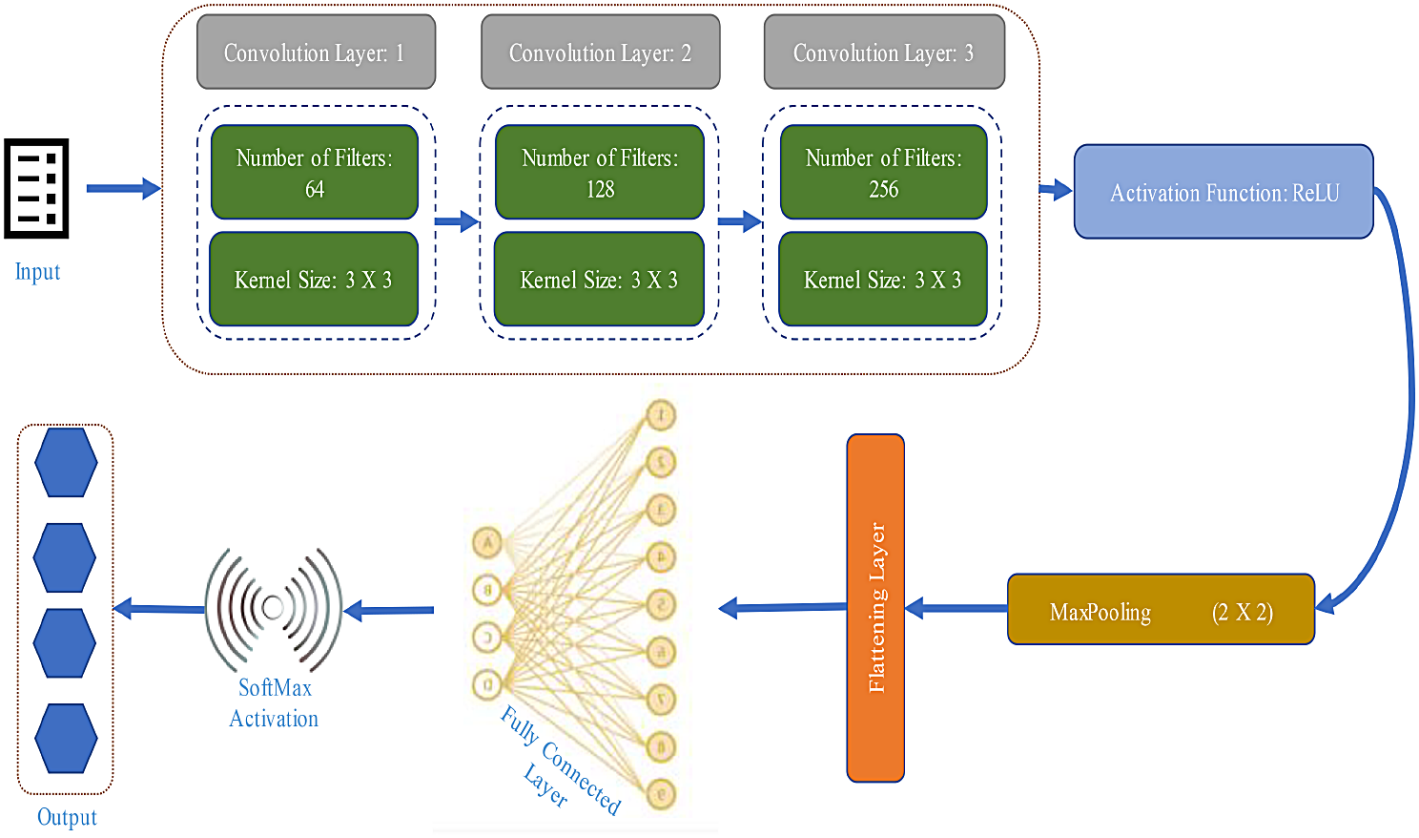
Proposed architecture of CNN model used for classification of diseases

The output layer will classify the input sensor data into various cardiorespiratory conditions such as normal sinus rhythm, bradycardia, tachycardia, etc. The proposed CNN model establishes robustness in processing data gathered from sensors deployed on human body for measuring cardio respiratory diseases by discerning intricate patterns and extracting discriminative features important for accurate classification of cardiorespiratory conditions. Figure 9 describes the architecture of CNN model.

#### Hyperparameter tuning and optimization

The CNN model accuracy and its prediction power changes with the change in it inter structure. Each time a value in the parameters changes it make the whole process. The Hyperparameter tuning is a crucial step in developing a high-performing CNN model. In the proposed study we focused on tuning key hyperparameters such as kernel sizes, number of filters, and learning rates to enhance the model’s accuracy and generalization capabilities. The hyperparameter considered as described below in Table 3.

**Table 3:** The hyperparameters considered during tuning

Sno	Parameter	Description
1	Kernel Size	The size of the convolutional filters.
2	Number of Filters	The number of convolutional filters in each layer.
3	Learning Rate	The step size for the optimizer.

**Fig. 10:**
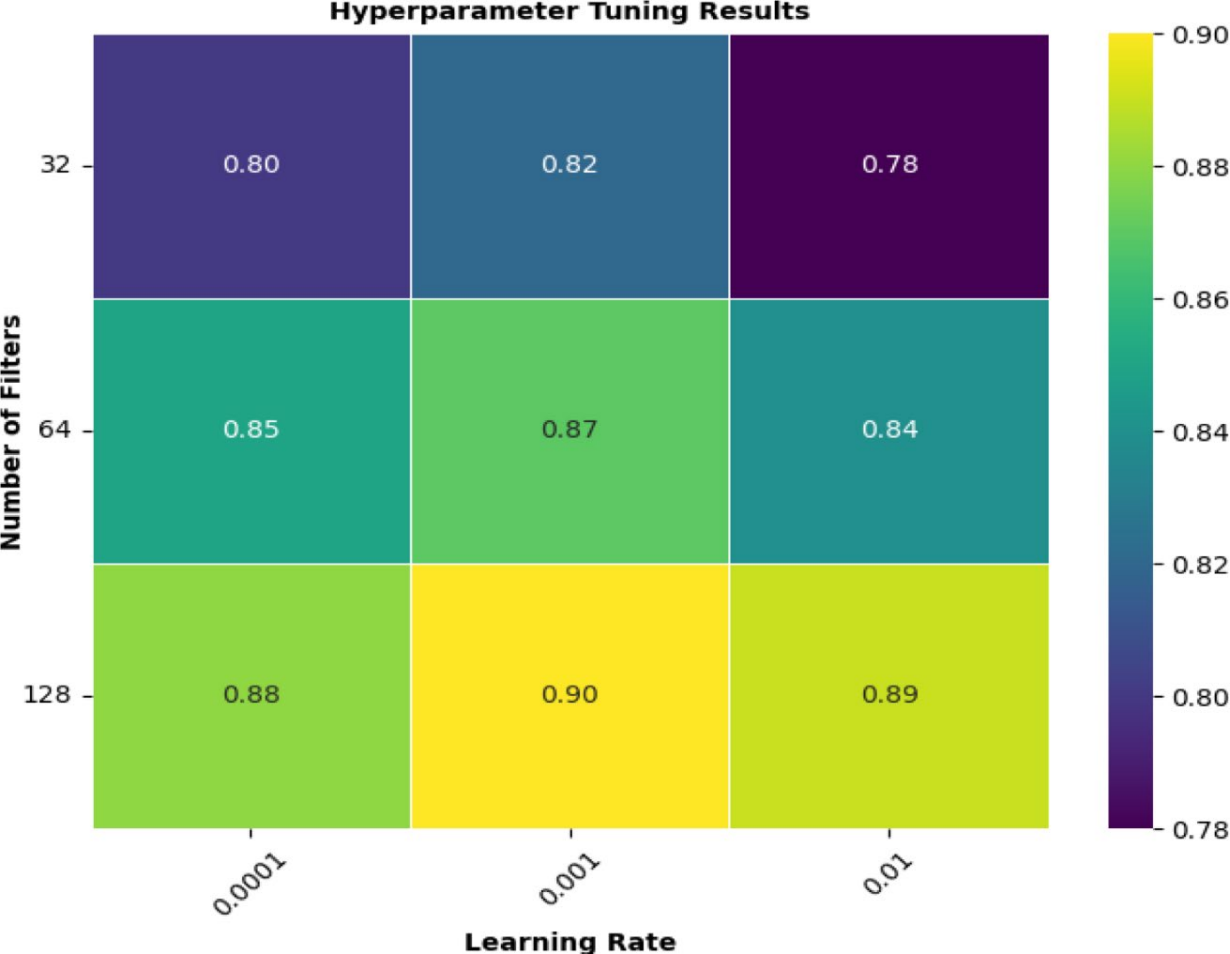
Visualization of Hyperparameter Tuning Results

To identify the best combination of hyperparameters a grid search is performed over a predefined range of values for each hyperparameter. The grid search was conducted using cross-validation to ensure robust performance metrics. First is the Kernel Sizes, the study is evaluate using kernel sizes of [3 × 3, 5 × 5, 7 × 7]. Smaller kernels capture fine details while larger kernels capture broader patterns. We found that a kernel size of 5 × 5 provided the best balance between detail and generalization. Second is the number of filters. The number of filters was varied across [32, 64, 128]. Increasing the number of filters allows the model to learn more complex features but also increases computational cost. Our experiments showed that 128 filters in the first convolutional layer achieved the highest accuracy without significantly increasing computation time. Third one is Learning Rates. This study use Learning rates of [0.0001, 0.001, 0.01]. A learning rate of 0.001 provided the best convergence speed and stability. The following heatmap visualizes the performance metrics for different combinations of hyperparameters, helping to identify the optimal values. Visualization of Hyperparameter Tuning is described below in Fig. 10.

## Evaluation, results & discussions

**Fig. 11:**
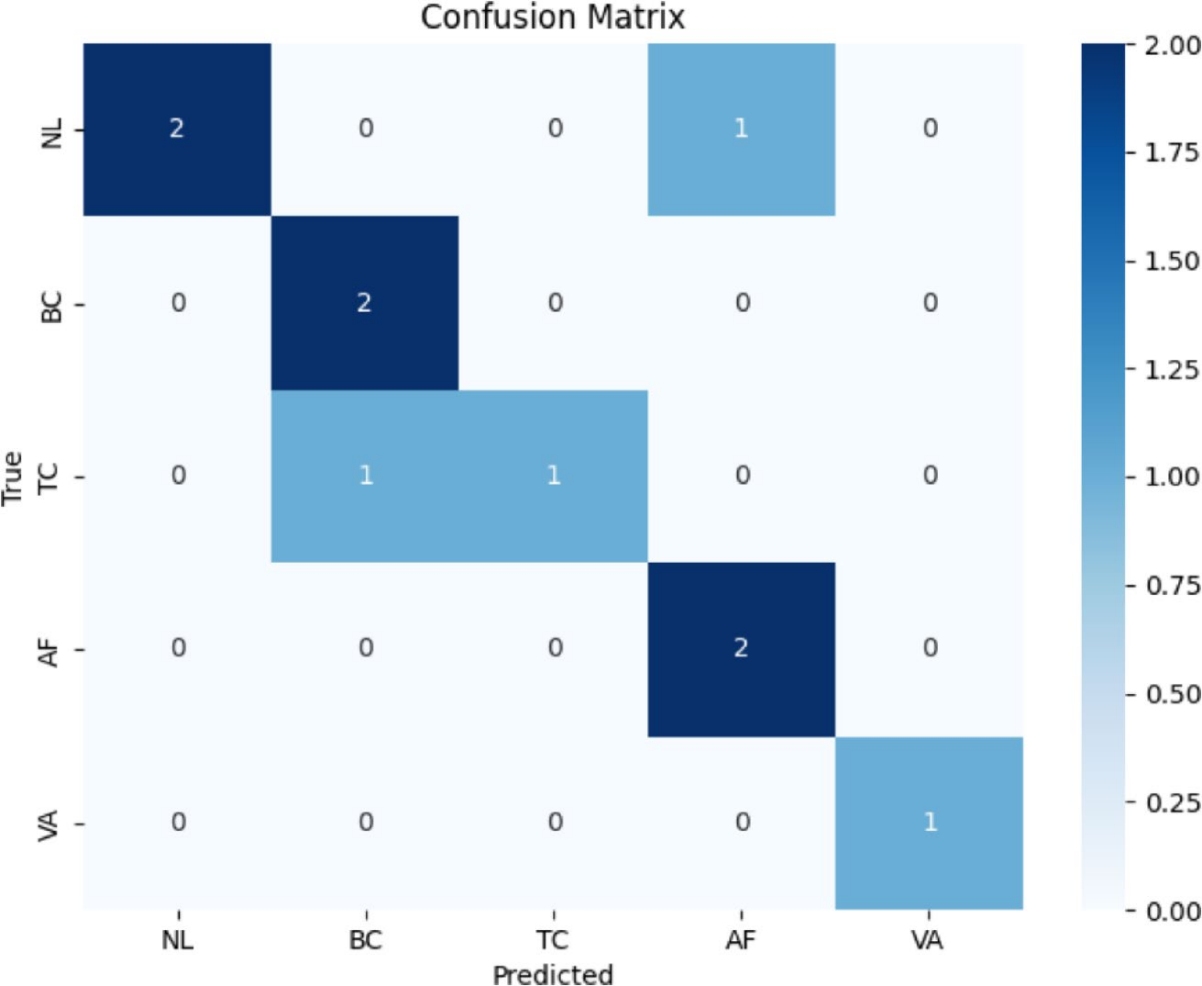
Confusion Matrix of the proposed study

The output of the CNN model determines the correct and incorrect instances identified from the data collected by sensor nodes. As the number of instances related to cardiorespiratory health increases or medical care is required, necessary steps are taken like hospital shifting or home care etc. If a patient’s condition is such that he can be treated at home the care is provided in the same place without shifting him to the hospital. This is significantly helpful in reducing the load on hospitals during pandemics. Furthermore, it assists in identifying the intensity of the pandemic. In all this process performance of the model is of vital importance because it defines the quality of the process in the identification and classification of diseases. In order the evaluate the performance of the proposed study it is compared with traditional methods for identifying cardiorespiratory diseases. A simulation study is used for comparison of the studies. To determine how many positive results and classes the proposed model identifies confusion matrix is utilized. It gives an account of the extrapolation of the values when they are considered from the model when it is likened to the original one. This is beneficial to the understanding that the current model provides the ability to properly classify events and establish possible ways of enhancing the model. It is an n x n matrix where the rows denote the actual class and columns indicate the predicted class across the instances. Every cell of the matrix or contingency matrix holds the number of conformities indicating the frequency of occurrences of the actual/ true class and the predicted class. Elements on the diagonal of the matrix are the ones where the actual class label is the same as the predicted class label, whereas elements other than the diagonal are where the actual class label is different from the one predicted. The classes that the confusion matrix represents are as follows and these classes are four in total. The first one is True Positives (TP), which depict Instances, which is of a particular class and have been correctly classified as belonging to that class by the model. The second is True Negative (TN) in which observations which certainly do not belong to a particular class are correctly rejected by the model. The third one is called False Positives (FP), which represents mislabeled instances that indeed are not part of a given class but the model defines them to be in a particular class. The last type is False Negatives (FN), which are instances that are in one class but are classified as negative to the class by our model. The confusion matrix of the above-said study has been described below in Fig. 11.

**Fig. 12:**
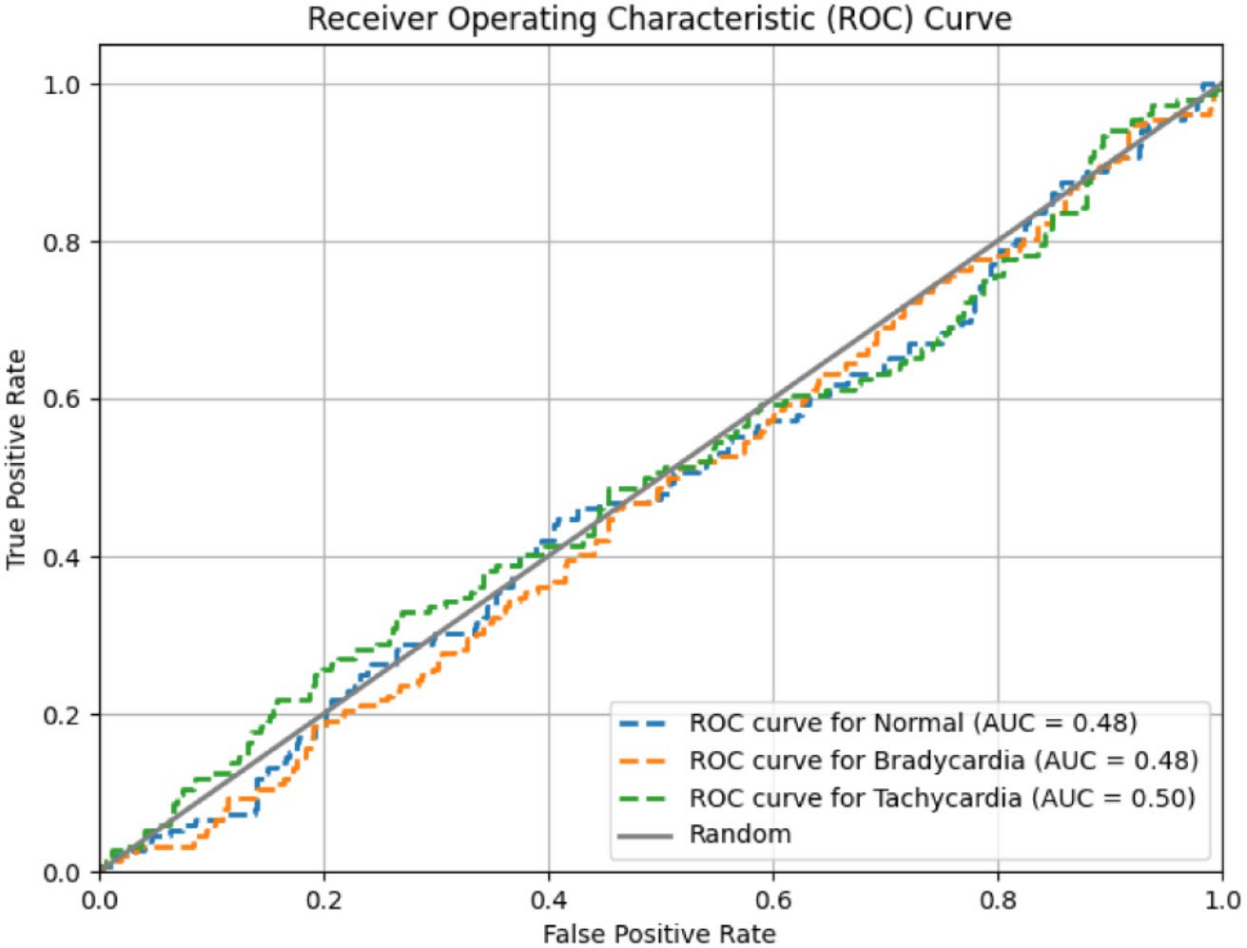
ROC Curve showing tradeoff between different classes

To ensure efficient performance of the classifier and the model trade-off should be established between true positive rate and false positive rate across different threshold values. The ROC curve is used to illustrate it and show the tradeoff. In the proposed study the CNN model predicts various health conditions using different class labels. Each class label represents a different health condition. The curve helps assess the model’s ability to distinguish between different health conditions accurately. A higher area under the ROC curve (AUC) indicates better overall performance of the model in distinguishing between different health conditions. Figure 12 depicts the ROC curve for the true positive and false positive values of the said study.

Together, the ROC curve and confusion matrix provide comprehensive insights into the classification performance of the CNN model in predicting different health conditions based on sensor data. Different performance metrices used for evaluation of the model are described below.

### Accuracy

To measure the performance of classification model utilized in the proposed study accuracy is used as a performance metric. It is an important metric that plays a crucial role in the evaluation of classification models because of its property to directly impacts the reliability and effectiveness of the proposed approach. In this work accuracy defines how well the developed CNN model can correctly classify instances into their respective classes and cardiorespiratory conditions. It can be calculated mathematically using the following Eq. 

20 $$\:Accuracy\:=\frac{TP+TN}{TP+FP+TN+FN}\:\:\:$$

**Fig. 13:**
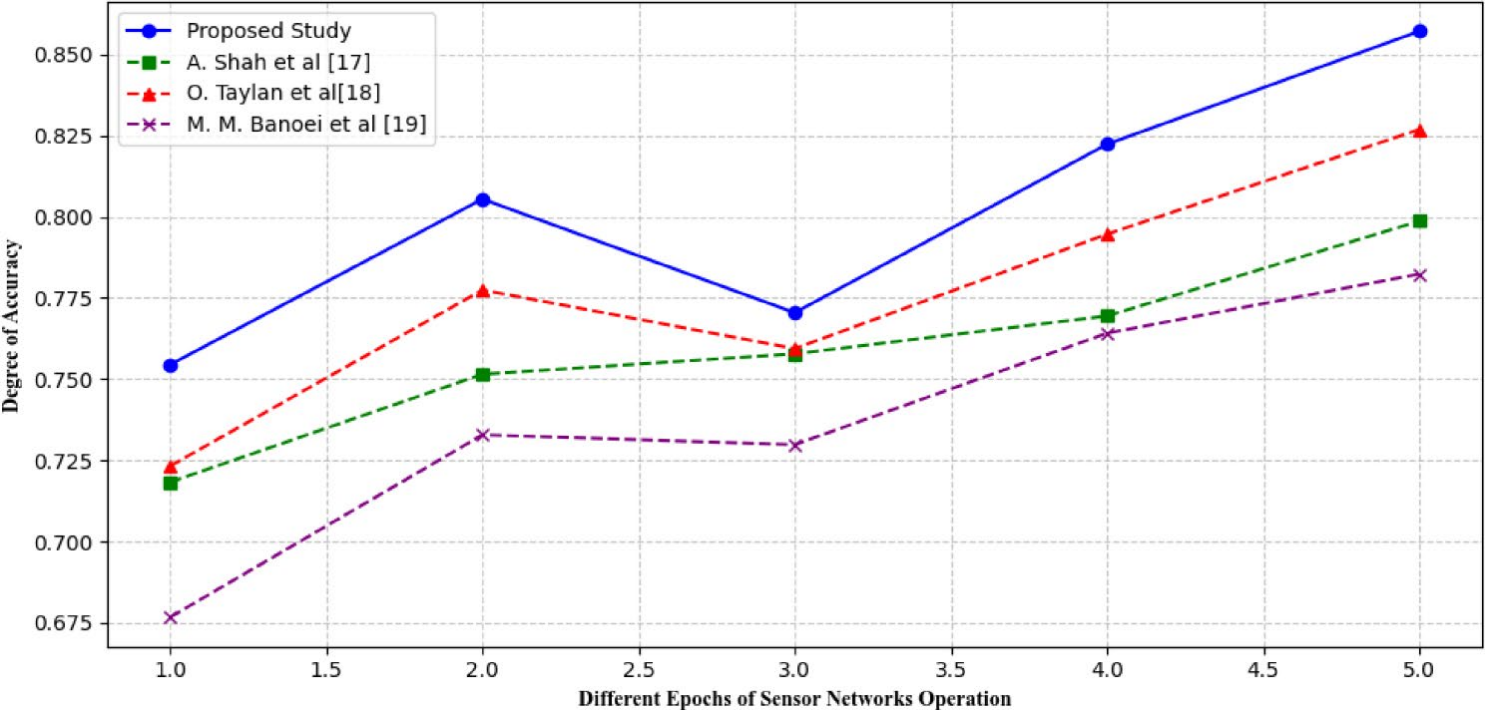
Comparison of different studies in terms of Accuracy

Where TP shows true positive rates, *TP* denote True positive, *TN* represent True Negative, *FP* depict False positive and *FB* is used for False positive. Achieving a high accuracy is essential for ensuring the model’s ability to make accurate predictions. A high value of accuracy metric implies that the model can effectively differentiate between different classes of conditions. The accuracy of the proposed model is contrasted with the accuracy of several alternative models. Figure 13 shows the accuracy values of different studies conducted for early detection of cardiorespiratory diseases.

### Sensitivity

The term sensitivity is defined as the measure of the proportion of actual positive cases that are correctly identified by the model. It is also termed as true positive rate or recall value. In the proposed study during different epochs of data collection sensitivity quantifies the ability of the model to correctly detect instances of specific cardiorespiratory conditions such as atrial fibrillation, ventricular arrhythmia, respiratory distress. A high sensitivity indicates that the model is effective at capturing true positive cases which minimize the number of false negative cases. The cases where model incorrectly predicts a negative outcome for a positive case. This performance metric is very helpful in cases where missing a positive case can have serious consequences such as failing to diagnose a life-threatening condition. To gauge the degree of sensitivity the dataset is typically divided into two groups i-e positive cases (instances of the condition being studied) and negative cases (instances without the condition). It is mathematically calculated using the following Eq. 

21 $$\:Sensitivity\:=\:\frac{TP}{(TP\:+\:FN)}$$

**Fig. 14:**
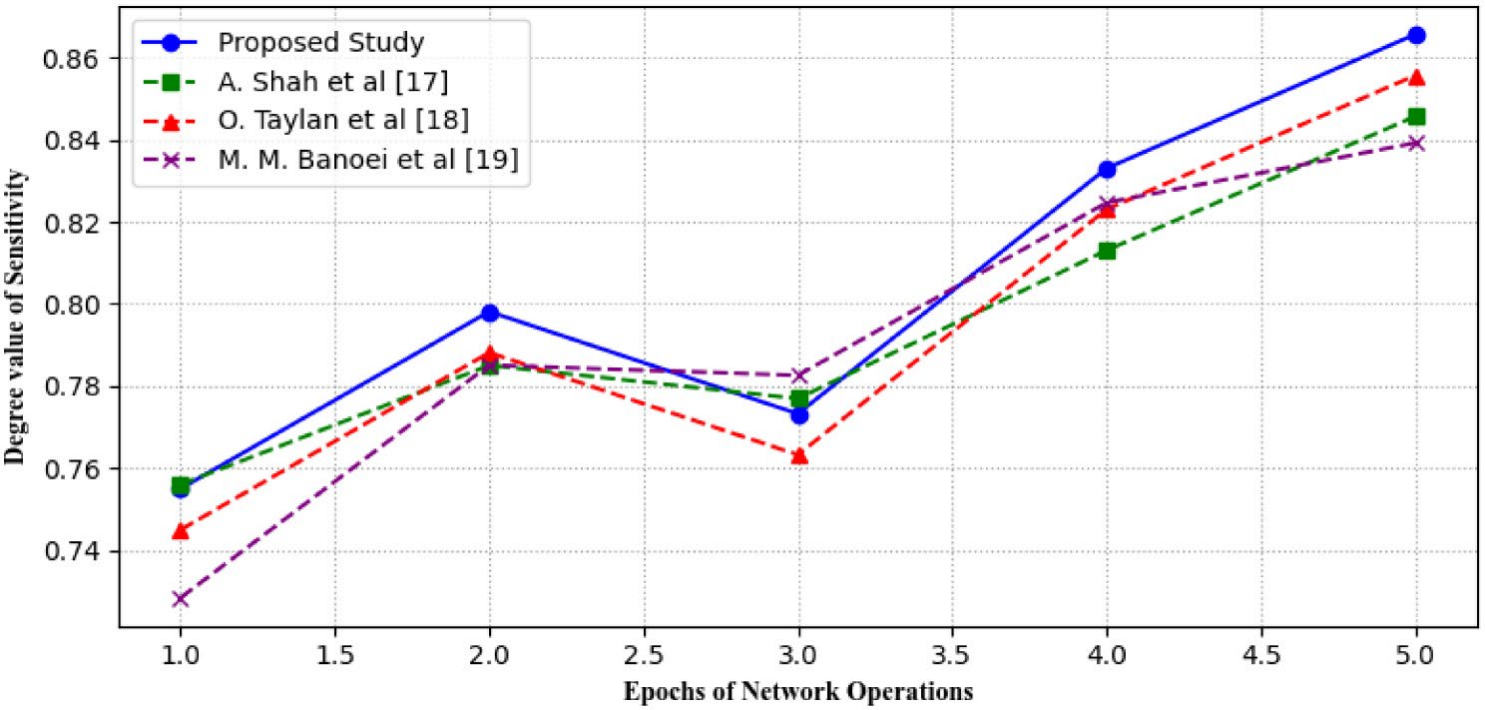
Comparison of different studies in terms of Sensitivity

It is calculated as the ratio of true positive predictions to the total number of positive cases in the dataset. Figure 14 depicts the graphical representation of sensitivity for the proposed study where the x-axis represents different thresholds or decision points used by the model while the y-axis represents the sensitivity value. It is also important in understanding the trade-off between sensitivity and specificity and determining the optimal threshold for the model in clinical practice.

### Specificity

It is defined as the extent to which the model can classify correct negative cases or true negatives in a given set out of all actual negative cases. They determine the errors which can occur in the data analysis process. On false positives, the term used to refer to the number of times the test signals that the attribute is present when it is, in fact, absent is called specificity. Mathematically, it is calculated using the following Eq. 

22 $$\:Specificity==\frac{\text{T}\text{P}}{\text{T}\text{P}\:+\:\text{F}\text{N}}\:\:\:\:\:\:\:\:\:\:\:\:\:\:\:\:\:\:\:$$

**Fig. 15:**
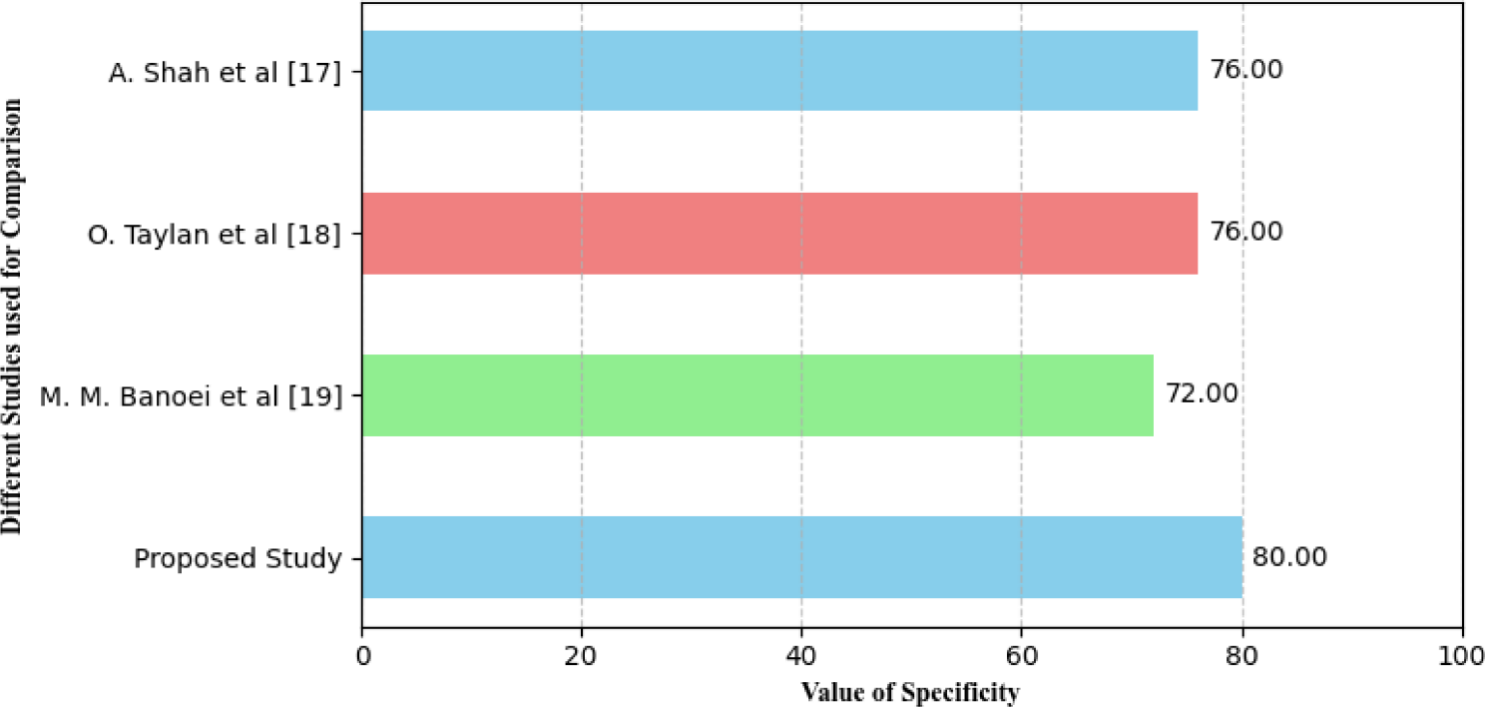
Specificity analysis of different models for identification of diseases

Where *TP* is True Positive and *FN* is False Negative. Specificity is good and when we get a high value of specificity then it means that the model is doing well in making wrong predictions in negative tests. This is probably true based on its negative predictions. On the other hand, we identify low specificity as meaning that in the negative class, the model is likely to misclassify many instances as belonging to the positive class. Figure 15 illustrates the values of calculated by the above-mentioned studies for the described studies.

### F1-Score

F score essentially is an evaluation of the performance of an operation or examination analysis that puts together at the same time, the concepts of precision and recall. Notably, it takes into account true positive (TP) together with false positive (FP) hence a balance of the two is provided. It is used to provide a degree of the relative performance of a classification model when the number of samples in a data set is skewed and it contains Different kinds of anomalies. Mathematically it can be represented using the following Eq. 

23 $$\:F1\:Score=\:\frac{2TP\:}{(2TP\:+\:FP\:+\:FN)\:\:\:}\:\:\:\:\:\:\:\:\:\:$$

**Fig. 16:**
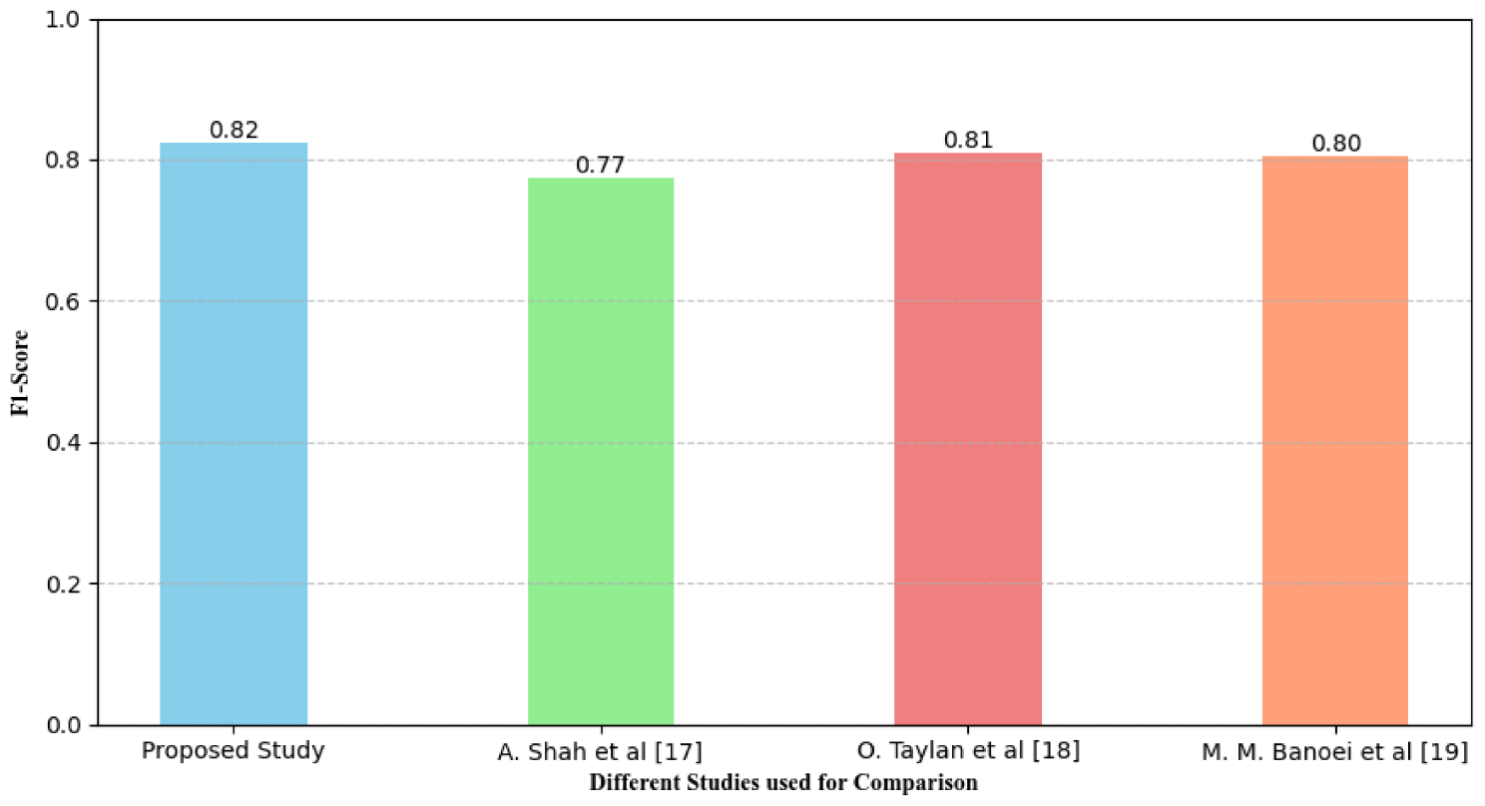
Specificity analysis of different models for identification of diseases

Where *TP* denote True Positive, *FP* is False Positive and *FN* donate False Negative. Figure 16 demonstrates evaluation of different studies in terms of F1 score.

### Precision

Accuracy is defined therefore as the measure of the proportion of the cases that the model correctly predicted as positive cases. They are widely used as an important performance metric applied to assess the accuracy of the correct estimates and reduce the number of false positives as well. A high precision value thus stood for good performance of the model in terms of its general ability to estimate true positives while a low value depicted its inability the estimate the true positive rate among all the classifiers. In mathematical terms, it has been defined and can be expressed in the following equation and takes a value of 0. According to the study suggested above, the subject identification code would be 5128.

24 $$\:\:\:Precision=\:\frac{TP}{(TP\:+\:FP)\:}\:\:\:\:\:\:\:$$

**Fig. 17:**
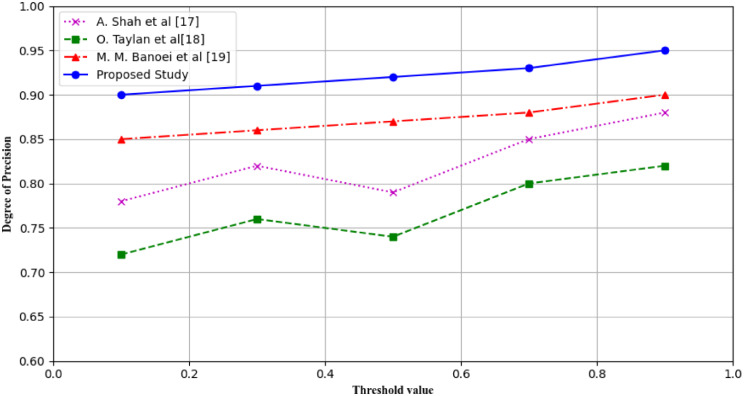
Precisions of different studies in classifying positive

Where TP represent True Positive and FN represent False Positive. The evaluation graph of precision measure of different research studies are conducted in Fig. 17.

### Precision-recall curve

**Fig. 18:**
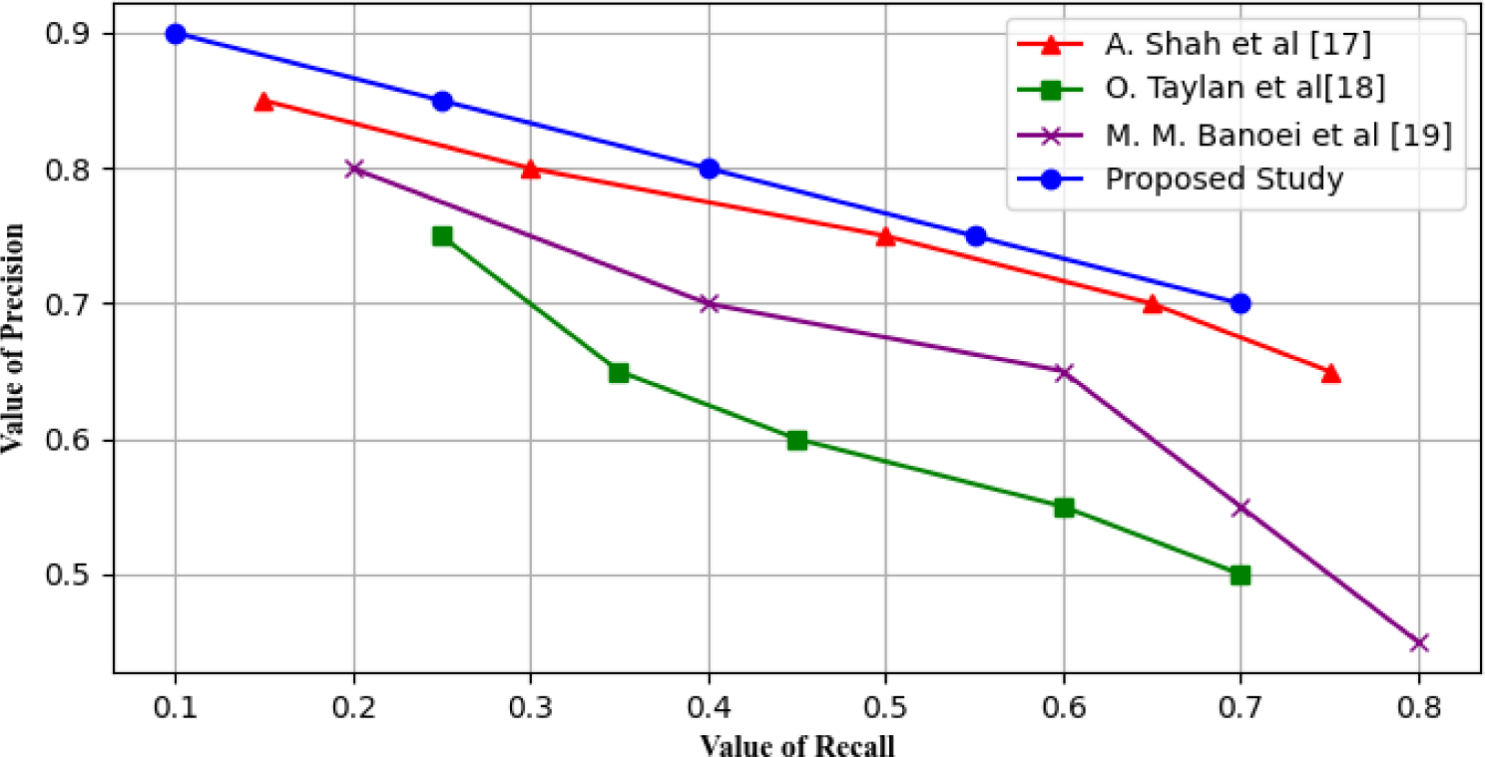
Precision recall curve during analysis of different studies

The tool used in graphical representation of the performances of the classification type of model is the precision-recall curve. Accuracy is concerned with the probability of results being correctly positive among all results that were predicted to be positive and recall focuses on the number of true positives that are identified by the model out of the actual positive values present in the data set. In this case, the curve that is produced from this study demonstrates the tendency of the precision and recall values as the threshold level is adjusted. It is used to illustrate the disorders in datasets in the course of analysis since it’s a means of handling imbalanced datasets where one class is most coveted than another. It evaluates the quality of the compromise between precision and recall which leads to the definition of proper threshold taking into consideration the desired outcome of the application. Figure 18 shown below clearly depicts that curve of the proposed study always lie above and to the left of other studies curve it means proposed study having higher precise and recall values which is better.

**Fig. 19:**
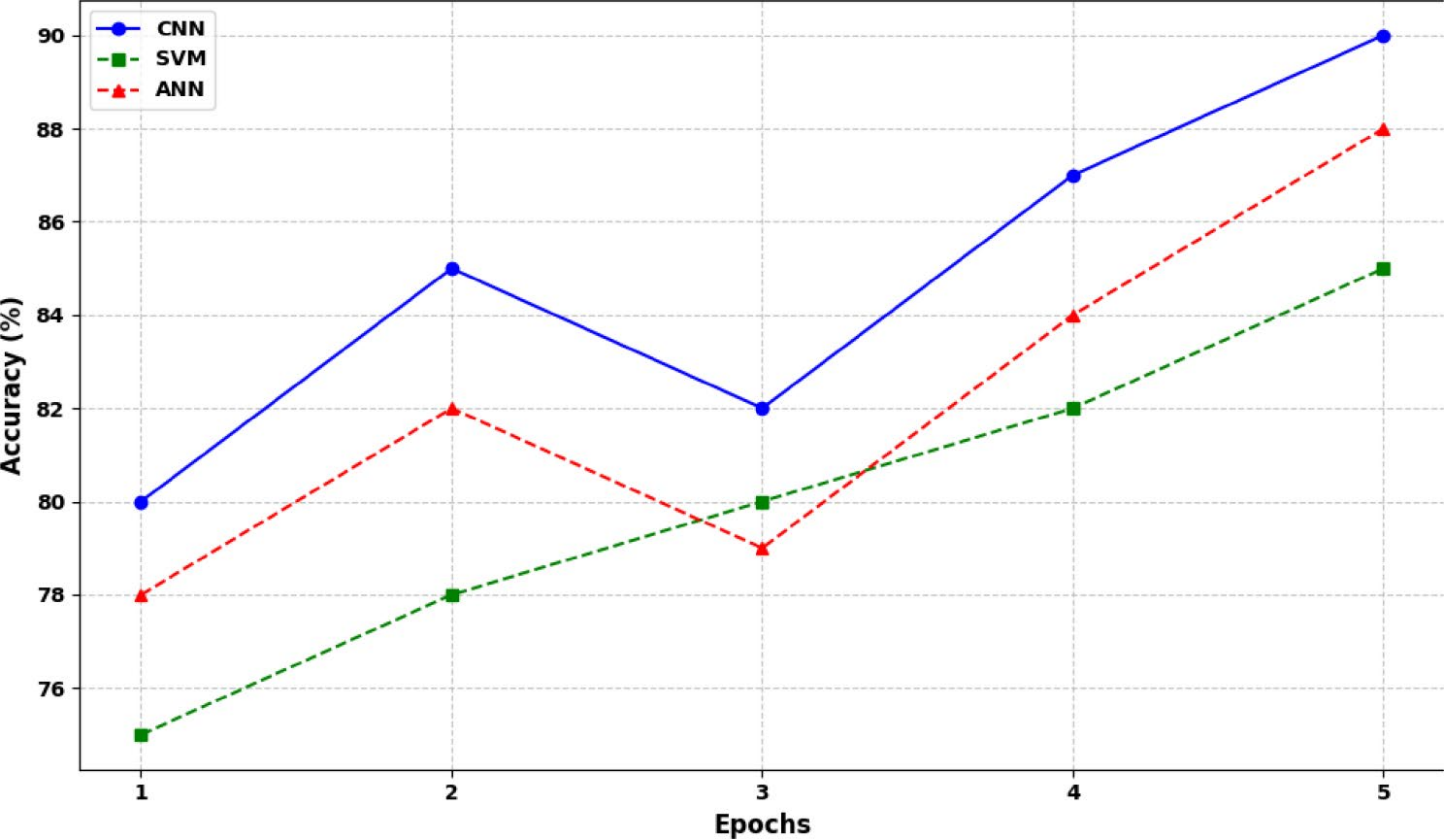
Performance comparison of state-of the art methods with the CNN

The proposed CNN model demonstrates superiority over Support Vector Machines (SVM) and Artificial Neural Networks (ANN) in several key aspects. SVMs rely on finding optimal hyperplanes in high-dimensional space while CNNs excel in learning hierarchical features directly from raw data particularly beneficial for tasks involving complex patterns and large datasets. CNNs’ ability to capture spatial and temporal dependencies through convolutional and pooling layers enhances their performance in tasks such as image classification, object detection, and medical diagnostics. In contrast, while ANNs like multi-layer perceptron’s (MLPs) excel in learning abstract representations, CNNs’ specialized architecture for grid-like data and automatic feature learning provide advantages in tasks requiring intricate data relationships and pattern recognition. Empirical comparisons in Fig. 19 consistently shows CNNs’ superior performance in these domains affirming their efficacy in advancing state-of-the-art methodologies in detection of cardiorespiratory diseases.

**Fig. 20:**
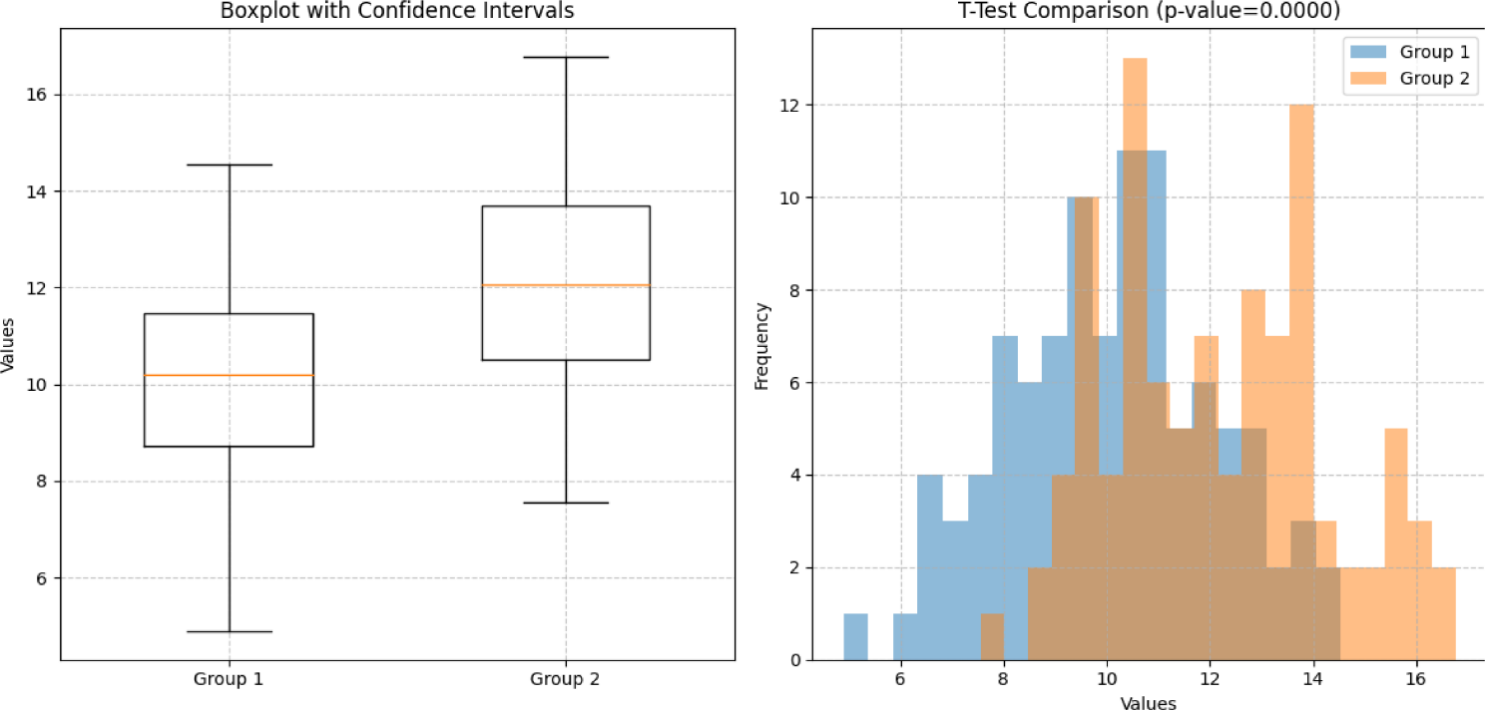
Statistical analysis of the data divided into Group 1 and Group 2

In order to determine the reliability of the results generated from the dataset. A statistical analysis of the data is conducted. The data used for training and testing is divided into two sub-groups. Each group has specific number of records. There is a comparison between two distinct groups of data labeled as Group 1 and Group 2. The first subplot in Fig. 19 utilizes a boxplot representation to visually summarize the distributions within each group. Each box in the plot denotes the interquartile range (IQR) of the data with a horizontal line indicating the median value. The whiskers extend to demonstrate the data range while individual points beyond the whiskers may represent outliers. This plot is designed to facilitate a comparative view of the data distributions between Group 1 and Group 2. Secondly, second subplot in the same plot depicts a histogram which illustrate the frequency distributions of data points in both groups. It provides a visual depiction of how data points are distributed across different values within each group. Additionally, a t-test comparison is performed to assess the statistical significance of the difference in means between Group 1 and Group 2. The p-value displayed in the chart quantifies the probability of observing the observed difference in means under the assumption that there is no true difference between the groups (null hypothesis). Lower p-values indicate stronger evidence against the null hypothesis which suggest a more significant difference between the groups as depicted in Fig. 20.

**Fig. 21:**
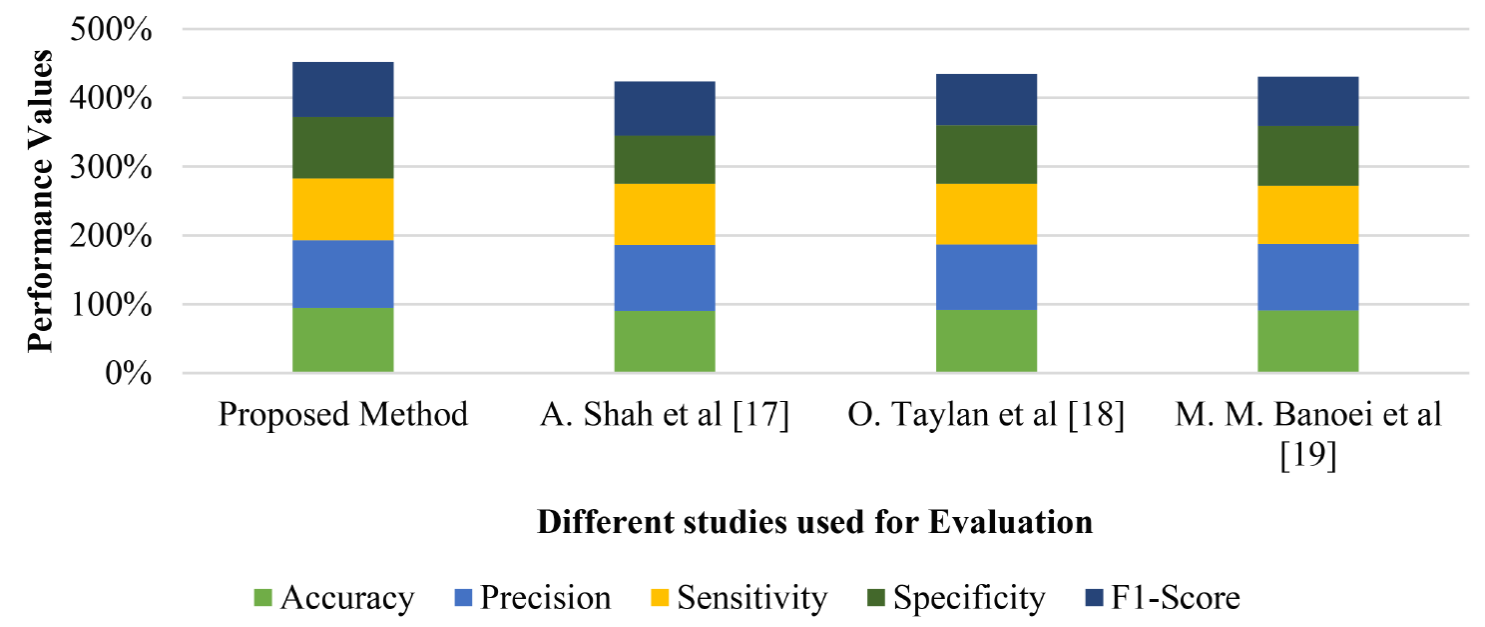
Performance evaluation of different studies used for diseases identification

The evaluation and comprehensive analysis of different studies used to determine cardiorespiratory diseases in pandemic using various performance metrics to demonstrate the better performance of the current study over its counterparts. The analysis of these performance metrics reveals that this process gives a holistic approach on the performance results of each model, enabling a knowledgeable choice of the right model alongside increasing understanding of the weaknesses and strengths of each model. In the following section, we depict the overall performance evaluation of the proposed study, which is presented in the form of a flowchart in Fig. 21.

## Conclusion

Early diagnosis of different diseases especially cardiorespiratory illness in a pandemic environment is of core importance. Timely identification can mitigate the load on health systems and decrease panic in society. This study proposed an IoT-based network in combination with the CNN model for the classification of health data specifically focusing on identifying different cardiac and respiratory conditions. To evaluate the performance rigorous experimentation of the proposed model has been committed, which shows that the proposed work consistently outperformed existing approaches in terms of accuracy, sensitivity, specificity, F1-score, and ROC curve. The results validate the effectiveness of the CNN model in accurately diagnosing various health conditions, which is beneficial for healthcare professionals in making timely and accurate diagnoses. While the study shows promising results there are several challenges for future research and improvement. It includes the expansion of the dataset to other health conditions and the incorporation of attention mechanisms and transfer learning for further enhancement of the model’s performance. Additionally, exploring simpler CNN architectures or employing model pruning techniques could enhance computational efficiency without compromising performance. Extending the framework to diverse populations and sensor configurations would validate its generalizability and broaden its applicability across different real-world scenarios addressing biases and limitations to ensure robust performance.

## Data Availability

To access the data that underpins the research findings, please get in touch with the designated author.
